# Mettl3-m^6^A-NPY axis governing neuron–microglia interaction regulates sleep amount of mice

**DOI:** 10.1038/s41421-024-00756-y

**Published:** 2025-02-04

**Authors:** Qihang Sun, Jinpiao Zhu, Xingsen Zhao, Xiaoli Huang, Wenzheng Qu, Xia Tang, Daqing Ma, Qiang Shu, Xuekun Li

**Affiliations:** 1 Children’s Hospital, School of Medicine, Zhejiang University, National Clinical Research Center for Child Health, Hangzhou, Zhejiang China; 2 The Institute of Translational Medicine, School of Medicine, Zhejiang University, Hangzhou, Zhejiang China; 3 Department of Rehabilitation, Perioperative and Systems Medicine Laboratory, Children’s Hospital, Zhejiang University School of Medicine, National Clinical Research Center for Child Health, Hangzhou, Zhejiang China; 4 Institute of Biotechnology, Xianghu Laboratory, Hangzhou, Zhejiang China; 5 Division of Anesthetics, Pain Medicine & Intensive Care, Department of Surgery and Cancer, Faculty of Medicine, Imperial College London, Chelsea and Westminster Hospital, London, UK; 6 Binjiang Institute of Zhejiang University, Hangzhou, Zhejiang China

**Keywords:** Cell signalling, Mechanisms of disease

## Abstract

Sleep behavior is regulated by diverse mechanisms including genetics, neuromodulation and environmental signals. However, it remains completely unknown regarding the roles of epitranscriptomics in regulating sleep behavior. In the present study, we showed that the deficiency of RNA m^6^A methyltransferase Mettl3 in excitatory neurons specifically induces microglia activation, neuroinflammation and neuronal loss in thalamus of mice. *Mettl3* deficiency remarkably disrupts sleep rhythm and reduces the amount of non-rapid eye movement sleep. We also showed that Mettl3 regulates *neuropeptide Y* (*NPY*) via m^6^A modification and *Mettl3* conditional knockout (cKO) mice displayed significantly decreased expression of NPY in thalamus. In addition, the dynamic distribution pattern of NPY is observed during wake-sleep cycle in cKO mice. Ectopic expression of Mettl3 and NPY significantly inhibits microglia activation and neuronal loss in thalamus, and restores the disrupted sleep behavior of cKO mice. Collectively, our study has revealed the critical function of Mettl3-m^6^A-NPY axis in regulating sleep behavior.

## Introduction

Sleep is a fundamental biological process and can be divided into two distinct states, i.e., rapid eye movement (REM) sleep and non-rapid eye movement (NREM) sleep^1^. Normal sleep-wake transition is critical for the health of animals and human being^1–3^. Sleep loss alters brain activity, synaptic structure and connections, and consequently induces neurological deficits including impaired learning, cognition, attention and emotion regulation^4–6^.

At molecular level, sleep is regulated by diverse mechanisms including genetics, DNA and histone modifications^7–11^. Sleep deprivation leads to the dysregulation of histone modification and histone deacetylase inhibitor suberoylanilide hydroxamic acid (SAHA) rescues the induced behavioral deficits^9,12,13^. Sleep deprivation also remarkably affects the expression of DNA methylation-related genes and circadian regulators^14–16^. Patients with REM sleep behavior disorder (RBD) display accelerated DNA methylation^17,18^. However, the function of RNA modification in regulating sleep behavior remains completely unknown.

*N*^6^-methyladenosine (m^6^A) is one of the most abundant and reversible RNA modifications, catalyzed by “writers” including Methyltransferase-like 3 (Mettl3), Mettl14, and Wilms tumor 1-associated protein (WTAP), removed by “erasers” including AlkB homolog 5 (Alkbh5) and Fat mass and obesity-associated protein (FTO), and recognized by YTH family proteins as well as HNRNPA2B1^19^. In the neuronal system, Mettl3-mediated m^6^A modification plays an essential role in regulating neurogenesis and neuronal development and is involved in neurological disorders^19–24^, but its function in regulating sleep behavior is not elucidated yet.

In the present study, we have shown that *Mettl3* deletion in CamkIIα^+^ neurons specifically leads to the activation of microglia and astrocytes, and induces neuroinflammation in the thalamus of mice. Activated microglia display phagocytic ability leading to neuronal loss. In addition to the reduced food intake, body weight, brain size and survival rate, *Mettl3* conditional knockout (cKO) mice display impaired sleep behavior, including the altered sleep-wake transition and the reduced amount of NREM sleep. Mechanistically, we have found that *neuropeptide Y* (*NPY*) mRNA is m^6^A-modified by Mettl3 and the expression of NPY is remarkably decreased in cKO mice. Ectopic NPY expression inhibits neuroinflammation and restores the disrupted sleep of cKO mice. Collectively, our study has revealed the important function and underlying mechanisms of Mettl3-mediated m^6^A modification in regulating sleep behavior.

## Results

### *Mettl3* deficiency specifically in CaMKIIα^+^ neurons reduces m^6^A levels in brain regions

To examine the function of Mettl3 in regulating the homeostasis of central nervous system, we first generated excitatory neuron-specific *Mettl3* cKO mice (*Mettl3*^*flox/flox*^*;CaMKIIα-Cre*, hereafter cKO) and littermate *Mettl3*^*flox/flox*^ mice (hereafter Ctrl) by crossing *Mettl3*^*flox/flox*^ mice with *Mettl3*^*flox/+*^*;CaMKIIα-Cre* mice (hereafter Het). To verify the knockout efficiency of *Mettl3*, we performed immunofluorescence staining and observed that the intensity of Mettl3 was dramatically decreased in several brain regions, including the thalamus, retrosplenial cortex and hippocampus of cKO mice compared to Ctrl mice at the age of 4 months (Supplementary Fig. S1a). mRNA and protein levels of Mettl3 were also significantly decreased in the thalamus, retrosplenial cortex and hippocampus of cKO mice compared to Ctrl mice at the age of 4 months (Supplementary Fig. S1b–j). Consistently, dot blot assay results showed that the level of m^6^A was significantly decreased in the thalamus, retrosplenial cortex and hippocampus of cKO mice compared to Ctrl mice at the age of 4 months (Supplementary Fig. S1k–p). The percentages of Mettl3^+^Iba1^+^ microglia, Mettl3^+^Gfap^+^ and Mettl3^+^S100β^+^ astrocytes showed no difference in the thalamus between Ctrl and cKO mice at the age of 2 months (Supplementary Fig. S1q–v). Together, cKO mice display *Mettl3* deficiency specifically in neuronal cells.

### *Mettl3* deficiency in CaMKIIα^+^ neurons specifically induces gliosis and neuroinflammation in thalamus

**Fig. 1:**
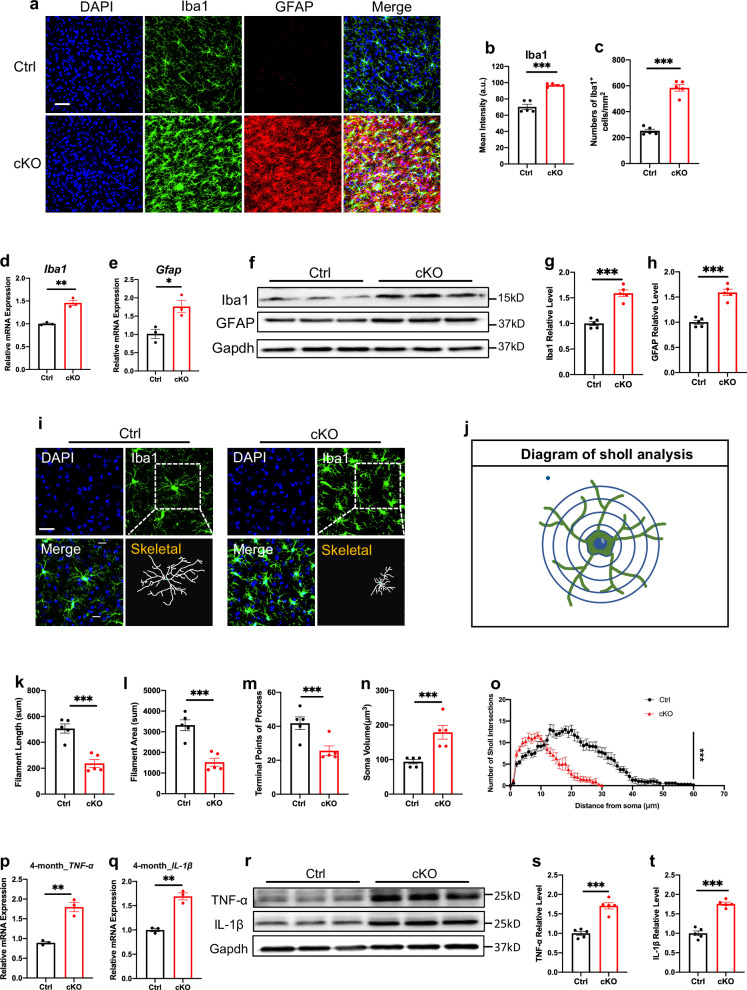
Neuronal *Mettl3* loss specifically induces neuroinflammation in thalamus. **a** Representative images of Iba1 and GFAP immunofluorescence staining in the thalamus of 4-month-old Ctrl and cKO mice. Scale bar, 50 μm. **b** Quantification of the fluorescence intensity of Iba1 in **a**. *n* = 5 mice for each group, and three sections were picked from each animal. Data are presented as mean ± SEM. Unpaired Student’s *t*-test, ***P < 0.0001. **c** Quantification of the number of Iba1^+^ cells in **a**. *n* = 5 mice for each group, and three sections were picked from each animal. Data are presented as mean ± SEM. Unpaired Student’s *t*-test, ***P < 0.0001. **d**, **e** qRT-PCR of *Iba1* (**d**) and *Gfap* (**e**) mRNA expression in the thalamus of 4-month-old mice. *n* = 3 independent experiments for each group. Data are presented as mean ± SEM. Unpaired Student’s *t*-test, *P < 0.05, **P < 0.001. **f**–**h** Western blot (**f**) and quantification results of Iba1 (**g**) and GFAP (**h**) levels in the thalamus of 4-month-old Ctrl and cKO mice. *n* = 5 mice for each group. Data are presented as mean ± SEM. Unpaired Student’s *t*-test, ***P < 0.0001. **i** Representative images of Iba1 immunofluorescence staining in thalamus regions of 4-month-old Ctrl and cKO mice for the 3D reconstruction of microglia. **j** Diagram of Sholl analysis for microglia using Imaris v7.2.3 software. Scale bar, 20 µm. **k**–**n** Quantification of microglial filament length (**k**), filament area (**l**), terminal points of the process (**m**), soma volume (**n**) in **i**. *n* = 5 mice for each group, and three sections were picked from each animal. Data are presented as mean ± SEM. Unpaired Student’s *t*-test, ***P < 0.0001. **o** Sholl analysis of microglia in **i**. *n* = 5 mice for each group, and three sections were picked from each animal. Data are presented as mean ± SEM. Two-way ANOVA followed by Tukey’s post hoc analysis, ***P < 0.0001. **p**, **q** qRT-PCR of TNF-α (**p**) and IL-1β (**q**) mRNA expression in the thalamus of 4-month-old mice. *n* = 3 independent experiments for each group. Data are presented as mean ± SEM. Unpaired Student’s *t*-test, **P < 0.001. **r**–**t** Western blot (**r**) and quantification results of TNF-α (**s**) and IL-1β (**t**) levels in the thalamus of 4-month-old Ctrl and cKO mice. *n* = 5 mice for each group. Data are presented as mean ± SEM. Unpaired Student’s *t*-test, ***P < 0.0001.

We next examined whether the loss of *Mettl3* induces brain cell changes. Indeed, *Mettl3* deficiency induced remarkable gliosis in the thalamus, but not in other brain regions, including the cortex and hippocampus of cKO mice at the age of 4 months compared to Ctrl mice (Fig. 1a; Supplementary Fig. S2a). Both the immunofluorescent intensity of Iba1 and the numbers of Iba1^+^ cells were significantly increased in the thalamus of cKO mice (Fig. 1b, c). mRNA and protein levels of Iba1 and GFAP were also remarkably increased in cKO mice compared to Ctrl mice at the age of 4 months (Fig. 1d–h). Morphologic analysis with three-dimension (3D) reconstruction showed that microglia exhibited an ameboid “stout” shape, i.e., an activated state, including the decreased filament length and area, decreased number of terminal points of the process, and increased soma volume in the thalamus of cKO mice at the age of 4 months (Fig. 1i–o). Of note, mRNA levels and immunofluorescent intensities of Iba1 and GFAP in the cortex and hippocampus showed no significant difference between Ctrl and cKO mice at the age of 4 months (Supplementary Fig. S2b–o). In addition, mRNA and protein levels of inflammatory cytokines tumor necrosis factor α (TNF-α) and interleukin-1β (IL-1β) were significantly increased in thalamus of cKO mice (Fig. 1p–t), but showed no difference in cortex and hippocampus of Ctrl and cKO mice (Supplementary Fig. S2p–y). Collectively, these results suggest that *Mettl3* loss specifically induces gliosis and neuroinflammation in the thalamus.

### The activation of microglia is prior to the activation of astrocytes in thalamus of the *Mettl3*-deficient mice

Next, we determined the temporal process of gliosis. Immunofluorescence staining and Sholl analysis showed that the morphology of microglia began to exhibit the activated state from the age of 2.25 months in the thalamus of cKO mice (Supplementary Fig. S3a, b). Meanwhile, GFAP^+^ astrocytes were detected at 2.5 months of age and significantly increased at the age of 3 months in the thalamus of cKO mice (Supplementary Fig. S3a, c). Morphological analysis showed that GFAP^+^ astrocytes displayed the activated state, including increased filament length and area, terminal points of the process and soma volume from postnatal 2.5 months to 4 months (Supplementary Fig. S3d–h). We further performed immunofluorescence staining with GFAP and S100β antibodies. We found that S100β antibody well detected astrocytes in the thalamus and the numbers of S100β^+^ astrocytes showed no difference between Ctrl and cKO mice at distinct time points (Supplementary Fig. S3i, j). Collectively, these results suggested that *Mettl3* deficiency-induced microglia activation is prior to the activation of astrocytes in the thalamus of cKO mice.

### *Mettl3* deficiency leads to neuronal and synaptic loss in thalamus of mice

**Fig. 2:**
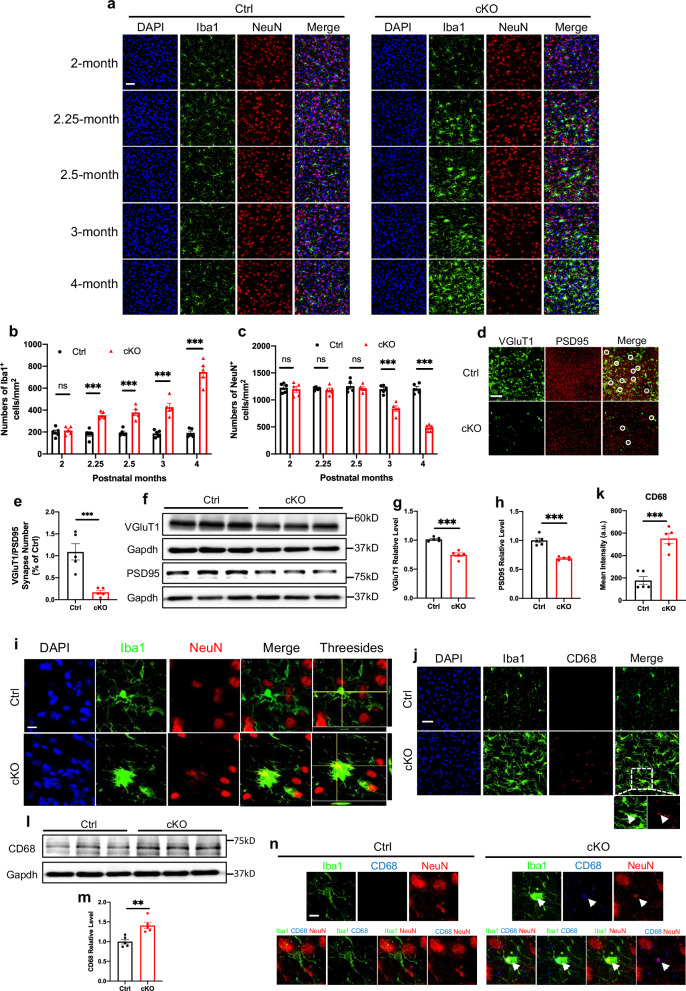
*Mettl3* deficiency induces the phagocytic activity of microglia and leads to neuronal and synaptic loss in the thalamus of mice. **a**–**c** Representative images of Iba1 and NeuN (**a**) immunofluorescence staining and quantification of the number of Iba1^+^ cells (**b**) and NeuN^+^ cells (**c**) in the thalamus of Ctrl and cKO mice at different ages. *n* = 5 mice for each group, and three sections were picked from each animal. Data are presented as mean ± SEM. Two-way ANOVA analysis followed by Tukey’s post hoc analysis; ns not significant; ***P < 0.0001. Scale bar, 50 µm. **d**, **e** Representative images of VGluT1 and PSD95 immunofluorescence staining (**d**) and quantification of the number of VGluT1^+^ PSD95^+^ synapse (**e**) in thalamus regions of 4-month-old Ctrl and cKO mice. *n* = 5 mice for each group, and three sections were picked from each animal. Data are presented as mean ± SEM. Unpaired Student’s *t*-test, ***P < 0.0001. Scale bar, 10 µm. **f**–**h** Western blot (**f**) and quantification results of VGluT1 (**g**) and PSD95 (**h**) levels in the thalamus of 4-month-old Ctrl and cKO mice. *n* = 5 mice for each group. Data are presented as mean ± SEM. Unpaired Student’s *t*-test, ***P < 0.0001. **i** Representative images of Iba1 and NeuN immunofluorescence staining in thalamus regions of 4-month-old Ctrl and cKO mice. Scale bar, 10 µm. **j**, **k** Representative images of Iba1 and CD68 immunofluorescence staining (**j**) and quantification of the fluorescence intensity of CD68 (**k**) in the thalamus of 4-month-old Ctrl and cKO mice. The white arrowhead indicates colocalization. Scale bar, 20 µm. *n* = 5 mice for each group, and three sections were picked from each animal. Data are presented as mean ± SEM. Unpaired Student’s *t*-test, ***P < 0.0001. **l**, **m** Western blot (**l**) and quantification results (**m**) of CD68 in the thalamus of 4-month-old Ctrl and cKO mice. *n* = 5 mice for each group. Data are presented as mean ± SEM. Unpaired Student’s *t*-test, **P < 0.001. **n** Representative images of Iba1, CD68 and NeuN immunofluorescence staining in the thalamus regions of 4-month-old Ctrl and cKO mice. The white arrowhead indicates colocalization. Scale bar, 10 µm.

Next, we examined the neuronal effects of *Mettl3* deficiency. Consistent with the activation of microglia, the number of Iba1^+^ microglia was significantly increased in the thalamus of cKO mice at postnatal 2.25 months and further increased until the age of 4 months (Fig. 2a, b). In addition, the number of NeuN^+^ neurons was significantly decreased in the thalamus of cKO mice at 3 months of age and further decreased until the age of 4 months (Fig. 2a, c; Supplementary Fig. S4a). Further, the presynaptic marker VGluT1 and postsynaptic marker PSD95 were both significantly decreased in the thalamus of cKO mice at the age of 4 months compared to Ctrl mice (Fig. 2d–h). Intriguingly, we did not find neuronal loss in the cortex and hippocampus, where microglia were not activated (Supplementary Fig. S4b–e). These results suggested the association between microglia activation and neuronal loss in the thalamus.

Next, we aimed to reveal the underlying mechanism for the neuronal and synaptic loss in the thalamus. We first performed western blot assays with the thalamic tissues of Ctrl and cKO mice at the ages of 3 and 4 months, when cKO mice showed neuronal loss. The levels of cleaved caspase-3 showed no difference between Ctrl and cKO mice at these two time points (Supplementary Fig. S4f–i). Given that activated microglia contribute to neuronal and synaptic loss via phagocytosis as reported previously^25–28^, we speculated whether activated microglia are involved in neuronal and synaptic loss in cKO mice under our experimental conditions. Immunofluorescence staining showed that fragmented NeuN signals localized in microglia in the thalamus of cKO mice at the age of 4 months, but not in Ctrl mice (Fig. 2i). Iba1^+^ microglia were also positive for phagocytosis marker CD68 (Fig. 2j, k). The protein level of CD68 was significantly increased in the thalamus of cKO mice compared to Ctrl mice (Fig. 2l, m). NeuN^+^ cellular debris was detected in CD68^+^Iba1^+^ microglia in the thalamus of cKO mice (Fig. 2n). Of note, the protein level of CD68 showed no change at the age of 2.25 months in the thalamus, when microglia were first activated and neurons were not lost in cKO mice (Supplementary Fig. S4j, k), and in cortex and hippocampus at the age of 4 months (Supplementary Fig. S4l–o). Collectively, these results suggested that activated microglia phagocytosed neurons leading to neuronal loss in the *Mettl3*-deficient mice.

### Neuronal *Mettl3* loss impairs sleep behavior

We next examined whether cKO mice displayed behavioral deficits. The level of Mettl3 significantly decreased in the thalamus of cKO mice from postnatal 2 weeks (Supplementary Fig. S5a–d) and significantly stunted growth of cKO mice was observed at the age of 4 months (Supplementary Fig. S5e). Relative to Ctrl and Het groups, cKO mice displayed decreased food intake from postnatal 12 weeks (Supplementary Fig. S5f), and reduced body weight from postnatal 12 weeks (Supplementary Fig. S5g). The brain weight was significantly decreased from the age of 12 weeks (Supplementary Fig. S5h). The survival curve showed that almost all cKO mice died around the age of 5 months (Supplementary Fig. S5i).

**Fig. 3:**
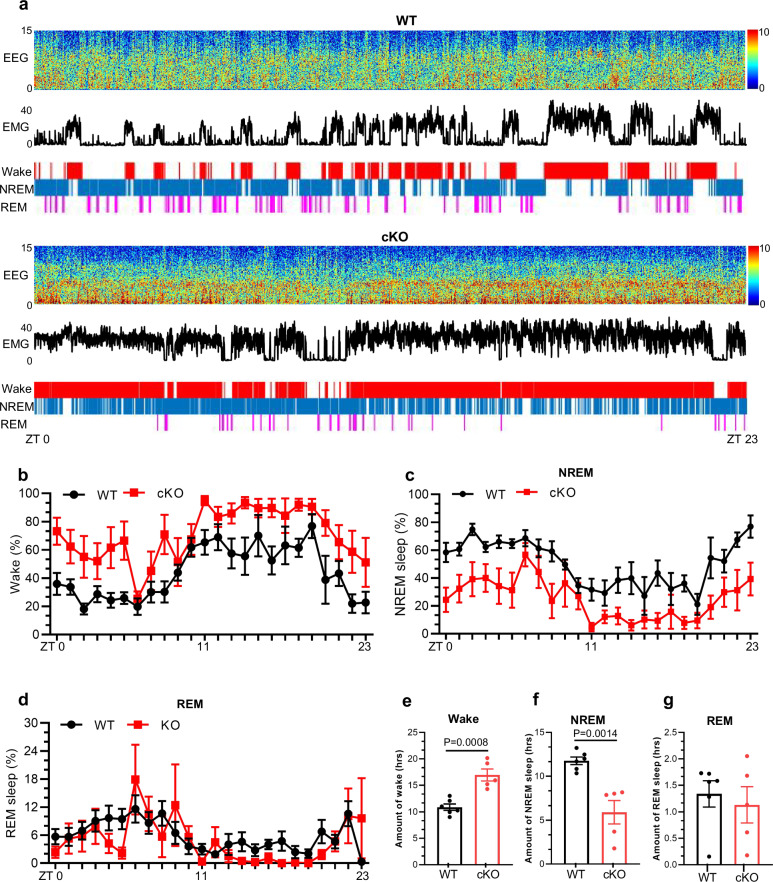
Neuronal *Mettl3* deficiency disrupts sleep behavior of mice. **a** Representative polysomnographic recording of 4-month-old Ctrl and cKO mice spanning from 8 AM (ZT 0) to 8 AM of the following day (ZT 23). Top, representative spectrogram of EEG; middle, EMG; bottom, brain states annotated including wake, NREM sleep and REM sleep were shown. ZT 0 indicated the start of the light period, and ZT 12 corresponded to the beginning of darkness. **b**–**d** The percentage of time spent in the wake (**b**), NREM sleep (**c**) and REM sleep (**d**) for each hour throughout 24-h recording. Data were analyzed using the two-way ANOVA with repeated measures. **e**–**g** The amount of time spent in the wake (**e**), NREM sleep (**f**) and REM sleep (**g**) during 24-h recording. *n* = 6 mice for Ctrl group and *n* = 5 mice for cKO group. ZT 0 indicates the start of the light period, and ZT 12 corresponds to the beginning of darkness. Data are presented as mean ± SEM. Unpaired Student’s *t*-test.

Recent studies have highlighted that the thalamus region plays a critical role in regulating wake-sleep behavior^29–31^. We then performed electroencephalogram (EEG) and electromyogram (EMG) recordings on 2- and 4-month-old Ctrl and cKO mice over a 24-h period, respectively (Fig. 3a; Supplementary Fig. S6a, b). Ctrl and cKO mice displayed similar proportion of wake state (F (1, 192) = 0.06617, *P* = 0.7973), NREM sleep (*F* (1, 192) = 0.4574, *P* = 0.04997) and REM sleep (*F* (1, 192) = 2.531, *P* = 0.1133) during the light-dark cycle, as well as the total amounts of three brain states at the age of 2 months (Supplementary Fig. S6e–g).

At the age of 4 months, the proportion of wake was significantly increased (*F* (1, 216) = 84.90, *P* < 0.0001) (Fig. 3a, b) and the proportion of NREM sleep was remarkably decreased in cKO mice relative to Ctrl mice (*F* (1, 216) = 99.19, *P* < 0.0001) (Fig. 3c). No significant difference was observed in REM sleep distribution between 4-month-old Ctrl and cKO mice (*F* (1, 216) = 1.589, *P* = 0.2088) (Fig. 3d). In addition, we quantified the total duration of brain states over 24-h period, and observed that the time spent in awake state was significantly increased in cKO mice (Fig. 3e) with a concomitant reduction of time spent in NREM sleep compared to Ctrl mice (Fig. 3f). The time for REM sleep showed no significant difference between Ctrl and cKO mice (Fig. 3g).

**Fig. 4:**
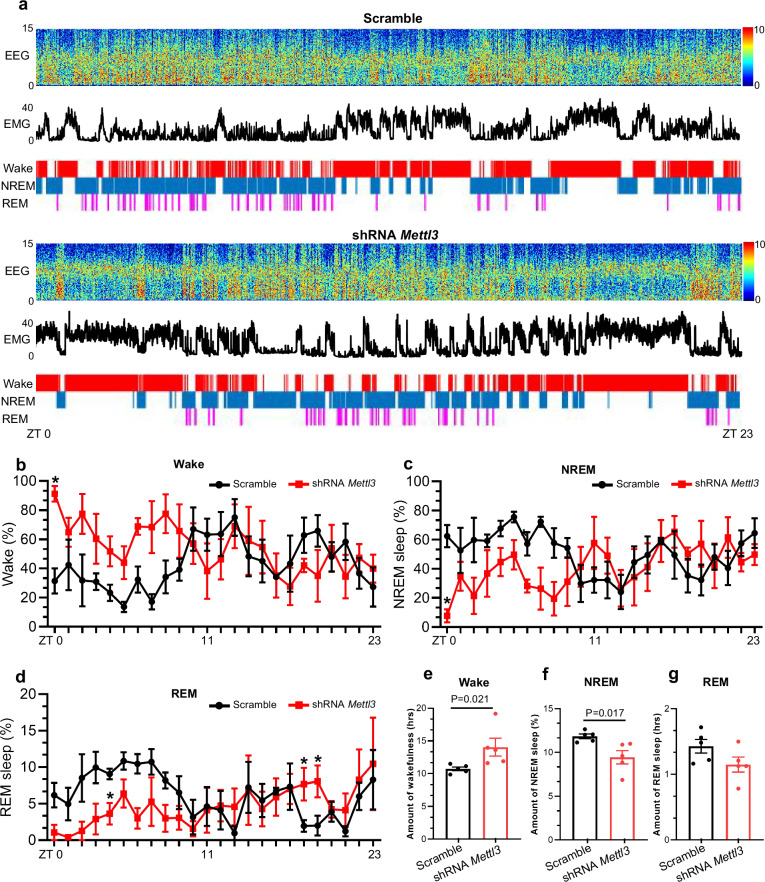
*Mettl3* acute knockdown disrupts sleep behavior of mice. **a** Representative polysomnographic recording of Ctrl group and *Mettl3* knockdown group mice spanning from 8 AM (ZT 0) to 8 AM of the following day (ZT 23). Top, representative spectrogram of EEG; middle, EMG; bottom, brain states annotated including wake, NREM sleep and REM sleep were shown. ZT 0 indicated the start of the light period, and ZT 12 corresponded to the beginning of darkness. **b**–**d** The percentage of time spent in wake (**b**), NREM sleep (**c**) and REM sleep (**d**) for each hour throughout the 24-h recording. Data were analyzed using the two-way ANOVA with repeated measures, *P < 0.05. **e**–**g** The amount of time spent in wake (**e**), NREM sl**e**ep (**f**) and REM sleep (**g**) across 24-h recording. *n* = 5 mice for each group. Data are presented as mean ± SEM. Unpaired Student’s *t*-test.

To verify the effects of thalamic Mettl3 on sleep behavior, we next performed *Mettl3* acute knockdown (KD) with AAV-shRNA against *Mettl3* in CaMKIIα^+^ neurons of the ventral posteromedial nucleus of the thalamus (VPM) of adult wild-type (WT) mice^32,33^ (Supplementary Fig. S7a, b), and ~80% of the total virus-infected area is VPM (Supplementary Fig. S7c). qRT-PCR and western blot assay showed that AAV-sh*Mettl3* significantly reduced the level of Mettl3 in the thalamus compared to that of Ctrl mice (scramble) (Supplementary Fig. S7f–h). Immunofluorescence staining and morphological analysis showed that *Mettl3* KD also induced activation of microglia and astrocytes (Supplementary Fig. S7i–p), and neuronal loss (Supplementary Fig. S7q, r). A 24-h recording of wake-sleep patterns was performed, showing that *Mettl3* KD led to a significant increase in the percentage of time in the awake state (*F* (1, 192) = 84.90, *P* = 0.0030) and a significant reduction in NREM sleep (*F* (1, 192) = 8.713, *P* = 0.0036) and REM sleep (*F* (1, 192) = 4.824, *P* = 0.0293) during the light-dark cycle (Fig. 4a–d). The total duration of the awake state was significantly increased in the KD group compared to Ctrl group (Fig. 4e), with a concomitant reduction of time spent in NREM sleep (Fig. 4f). The time for REM sleep showed no significant difference between Ctrl and KD mice (Fig. 4g). These results suggested that thalamic Mettl3 indeed regulates sleep behavior.

### *Mettl3* deficiency alters gene expression in thalamus

**Fig. 5:**
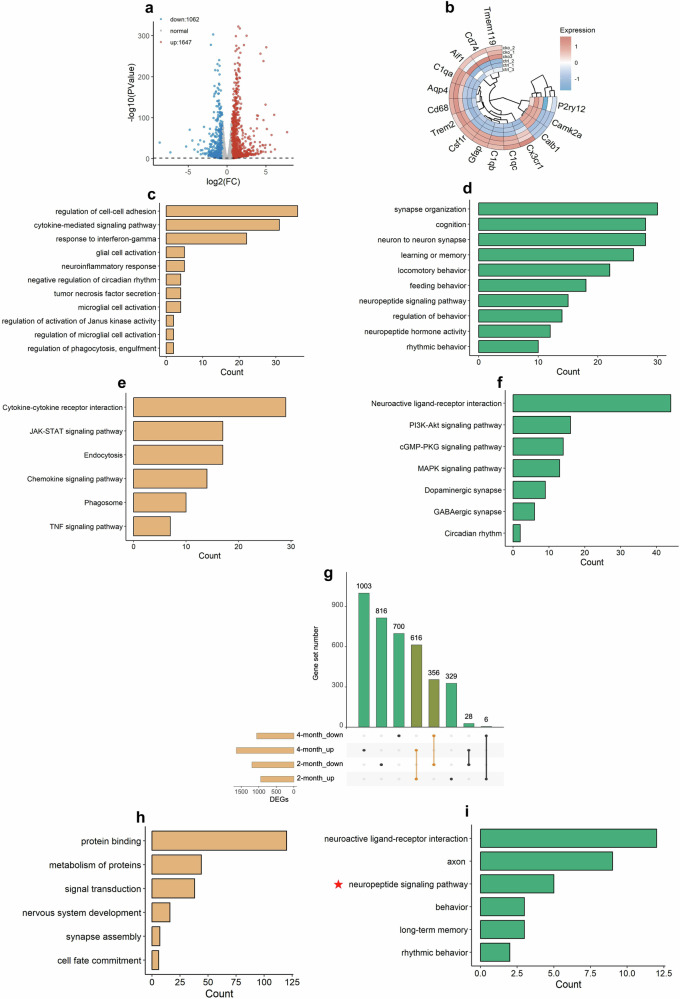
*Mettl3* deficiency alters gene expression in the thalamus of mice. **a** Volcano plot for DEGs in 4-month-old cKO mice compared to Ctrl mice. Three biological repeat samples of Ctrl and cKO mice were adopted for sequencing. Fold change = 1.5 and *P* = 0.05 were considered as significant. The list of DEGs can be found in Supplementary Table S3. **b** Relative gene expression levels of microglia markers of different functions and relative gene expression levels of astrocyte markers and neuron markers. **c**, **d** GO analysis of the upregulated (**c**) and downregulated (**d**) biological processes in the thalamus region of 4-month-old cKO mice compared with Ctrl mice. **e**, **f** KEGG analysis of the upregulated (**e**) and downregulated (**f**) genes in the thalamus region of 4-month-old cKO mice compared with Ctrl mice. **g** Overlapped DEGs between 2- and 4-month-old cKO mice. **h**, **i** GO analysis of the upregulated (**h**) and downregulated (**i**) overlapped DEGs in the thalamus of 2- and 4-month-old cKO mice. Red star in **i** denotes neuropeptide term identified in downregulated DEGs.

We next aimed to uncover the underlying mechanisms of how neuronal *Mettl3* loss induces microglia activation and sleep disruption. We first performed RNA-sequencing (RNA-seq) with thalamic tissues of 2- and 4-month-old Ctrl and cKO mice, respectively. In 2-month-old cKO mice, RNA-seq data analysis identified 2151 differentially expressed genes (DEGs) with 951 upregulated and 1200 downregulated (Supplementary Table S2). In 4-month-old mice, RNA-seq data analysis identified 2709 DEGs with 1647 upregulated and 1062 downregulated (Fig. 5a; Supplementary Table S2). The expression of microglia-associated genes, including *Aif1*, *Tmem119*, *Csf1r*, *Cx3cr1* and astrocyte-associated genes, including *GFAP* and *Aqp4* were significantly increased, but neuronal-associated genes, such as *CaMKIIα* and *Calb1* were significantly decreased (Fig. 5b). Of note, the expression of microglia activated state-related genes including *CD74*, *CD68*, *C1qa*, *C1qb* and *C1qc* were significantly increased, but microglia quiescent state-related gene *P2ry12* was significantly decreased in thalamus of cKO mice relative to Ctrl mice at the age of 4 months (Fig. 5b).

Gene ontology (GO) analysis showed that upregulated DEGs of 4-month-old mice were enriched for terms including neuroinflammation response and phagocytosis, and downregulated genes were enriched for terms including neuropeptide signaling pathway, feeding behavior and rhythmic behavior (Fig. 5c, d). Enrichment analysis of Kyoto Encyclopedia of Genes and Genomes (KEGG) pathways showed that upregulated DEGs were mainly enriched for cytokine activity and JAK-STAT signaling pathway (Fig. 5e), and downregulated DEGs were mostly enriched for neuroactive ligand–receptor interaction (Fig. 5f). GO analysis with the overlapped DEGs between 2- and 4-month-old cKO mice showed that downregulated DEGs were also enriched for neuropeptide signaling pathway (Fig. 5g, i), while the terms enriched by upregulated DEGs showed no good correlation with phenotypes observed in cKO mice (Fig. 5h). Collectively, these data suggested that *Mettl3* deficiency alters the expression of genes related to neuroinflammation and microglia activation.

### Neuronal *Mettl3* deficiency reduces NPY and activates the STAT3 pathway in the thalamus

**Fig. 6:**
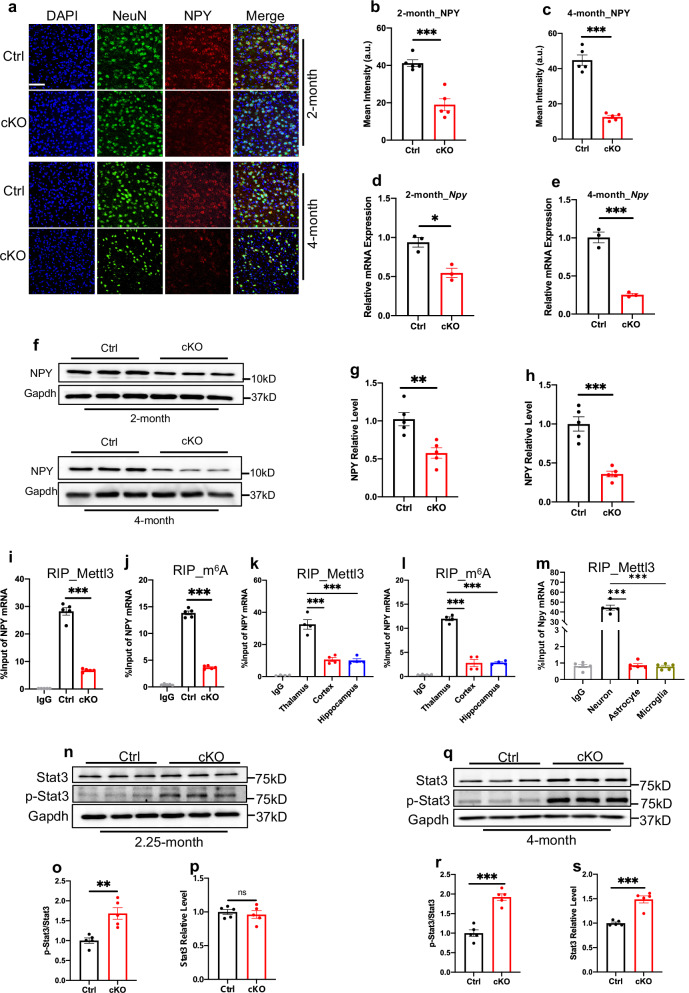
*Mettl3* deletion specifically reduces NPY and activates the STAT3 pathway in the thalamus of mice. **a**–**c** Representative images of NeuN and NPY (**a**) immunofluorescence staining and the quantification of the fluorescence intensity of NPY in the thalamus region of 2-month-old (**b**) and 4-month-old (**c**) Ctrl and cKO mice. *n* = 5 mice for each group, and 3 sections were picked from each animal. Data are presented as mean ± SEM. Unpaired Student’s *t*-test, ***P < 0.0001. Scale bar, 50 µm. **d**, **e** qRT-PCR of *NPY* mRNA expression in the thalamus of 2-month-old (**d**) and 4-month-old (**e**) mice. *n* = 3 indepe*n*dent experiments for each group. Data are presented as mean ± SEM. Unpaired Student’s *t*-test, *P < 0.05, ***P < 0.0001. **f**–**h** Western blot (**f**) and quantification results of NPY levels in the thalamus of 2-month-old (**g**) and 4-month-old (**h**) Ctrl and cKO mice. *n* = 5 mice for each group. Data are presented as mean ± SEM. Unpaired Student’s *t*-test, **P < 0.001, ***P < 0.0001. **i** Mettl3 RIP followed by qRT-PCR showed that the binding of Mettl3 to *NPY* mRNA was significantly decreased in the thalamus of cKO mice at the age of 4 months. *n* = 5 mice for each group. Data are presented as mean ± SEM. One-way ANOVA followed by Tukey’s post hoc analysis, ***P < 0.0001. **j** m^6^A-RIP followed by qRT-PCR showed that m^6^A modification of *NPY* significantly decreased in the thalamus of 4-month-old cKO mice. *n* = 5 mice for each group. Data are presented as mean ± SEM. One-way ANOVA followed by Tukey’s post hoc analysis, ***P < 0.0001. **k** Mettl3 RIP, followed by qRT-PCR, showed that the binding of Mettl3 to *NPY* mRNA was higher in the thalamus compared to the cortex and hippocampus of cKO mice. *n* = 4 mice for each group. Data are presented as mean ± SEM. One-way ANOVA followed by Tukey’s post hoc analysis, ***P < 0.0001. **l** m^6^A-RIP followed by qRT-PCR showed that the level of m^6^A modification of *NPY* was higher in the thalamus compared to the cortex and hippocampus of cKO mice. *n* = 4 mice for each group. Data are presented as mean ± SEM. One-way ANOVA followed by Tukey’s post hoc analysis, ***P < 0.0001. **m** Mettl3 RIP followed by qRT-PCR showed that the binding of Mettl3 to *NPY* mRNA was higher in neurons compared to astrocytes and BV2 microglia. *n* = 5 independent experiments for each group. Data are presented as mean ± SEM. One-way ANOVA followed by Tukey’s post hoc analysis, ***P < 0.0001. **n**–**p** Western blot (**n**) and quantification results of p-Stat3/Stat3 ratios (**o**) and Stat3 levels (**p**) in the thalamus of 2.25-month-old Ctrl and cKO mice. *n* = 5 mice for each group. Data are presented as mean ± SEM. Unpaired Student’s *t*-test; ns, not significant; **P < 0.001. **q**–**s** Western blot (**q**) and quantification results of p-Stat3/Stat3 ratios (**r**) and Stat3 levels (**s**) in the thalamus of 4-month-old Ctrl and cKO mice. *n* = 5 mice for each group. Data are presented as mean ± SEM. Unpaired Student’s *t*-test, ***P < 0.0001.

Previous studies have shown the critical function of NPY in neuroinflammation^34^. We then examined whether the downregulated NPY is involved in microglia activation and neuroinflammation induced by *Mettl3* deficiency. The intensity of NPY in the thalamus was significantly decreased in cKO mice compared to Ctrl mice at the ages of 2 and 4 months (Fig. 6a–c). mRNA and protein levels of NPY were also significantly decreased in thalamus of cKO mice compared to Ctrl mice of the same age (Fig. 6d–h), but immunofluorescence staining (Supplementary Fig. S8a–f), qRT-PCR (Supplementary Fig. S8g–j), and western blot (Supplementary Fig. S8k–p) of NPY showed no difference in cortex and hippocampus between Ctrl and cKO mice at the ages of 2 and 4 months.

Next, we explored the underlying mechanism for specific alteration of NPY in the thalamus. Immunofluorescence staining showed that Mettl3 and NPY were co-localized in CamKIIα^+^ neurons (Supplementary Fig. S8q). Western blot and qRT-PCR showed that NPY displayed the highest level in the thalamus compared to the cortex and hippocampus of mice (Supplementary Fig. S8r–t). RNA immunoprecipitation (RIP) with Mettl3 antibody followed by qPCR (RIP-qPCR) showed that the binding of Mettl3 with *NPY* mRNA was significantly decreased in the thalamus of cKO mice compared to Ctrl mice at 4 months of age (Fig. 6i). Consistently, m^6^A-methylated RNA immunoprecipitation followed by qPCR (MeRIP-qPCR) showed that the level of m^6^A modification on *NPY* mRNA was significantly decreased in the thalamus of cKO mice compared to Ctrl mice at 4 months of age (Fig. 6j). In addition, Mettl3 RIP-qPCR showed that the binding of Mettl3 with *NPY* mRNA was higher in thalamus compared to cortex and hippocampus (Fig. 6k). As a consequence, the level of m^6^A modification on *NPY* mRNA was higher in the thalamus compared to cortex and hippocampus (Fig. 6l). Mettl3 RIP-qPCR showed significantly higher binding of Mettl3 to *NPY* mRNA in cultured neurons compared to astrocytes and BV2 microglia (Fig. 6m). qRT-PCR showed that *NPY1r* had the highest expression compared to *NPY2r*, *NPY4r* and *NPY5r* in thalamus of 2-month-old WT mice (Supplementary Fig. S9a). Immunofluorescence staining and western blot showed that the expression of NPY1r was significantly increased in the thalamus of 4-month-old cKO mice compared to Ctrl mice (Supplementary Fig. S9b–h). Collectively, these results suggest that *NPY* mRNA in the thalamus is intensively m^6^A-modified by Mettl3.

Our RNA-seq data analysis showed that the upregulated DEGs are enriched for the JAK-STAT3 pathway, which plays important roles in neuroinflammation^35–38^. We observed that at the age of 2 months, when microglia were not activated, both the pSTAT3/STAT3 ratio and total STAT3 level were not altered in the thalamus of cKO mice relative to Ctrl mice (Supplementary Fig. S10a–c). At 2.25 months of age, when microglia began to be activated, but neurons were not lost, the pSTAT3/STAT3 ratio was significantly increased, while the total STAT3 level was not altered in the thalamus of cKO mice compared to Ctrl mice (Fig. 6n–p). At 4 months of age, when cKO mice displayed remarkable microglia activation, neuroinflammation and neuronal loss, both pSTAT3/STAT3 ratio and total STAT3 level were significantly increased in the thalamus of cKO mice compared to Ctrl mice (Fig. 6q–s). Of note, both total STAT3 and pSTAT3 levels showed no difference in the cortex and hippocampus of cKO mice compared to Ctrl mice at the age of 4 months (Supplementary Fig. S10d–i). Collectively, these results suggest that *Mettl3* deficiency specifically reduces NPY via downregulating its m^6^A modification and activates JAK-STAT3 signaling in the thalamus.

### Ectopic NPY expression inhibits microglia activation and restores the disrupted sleep behavior induced by *Mettl3* deficiency

**Fig. 7:**
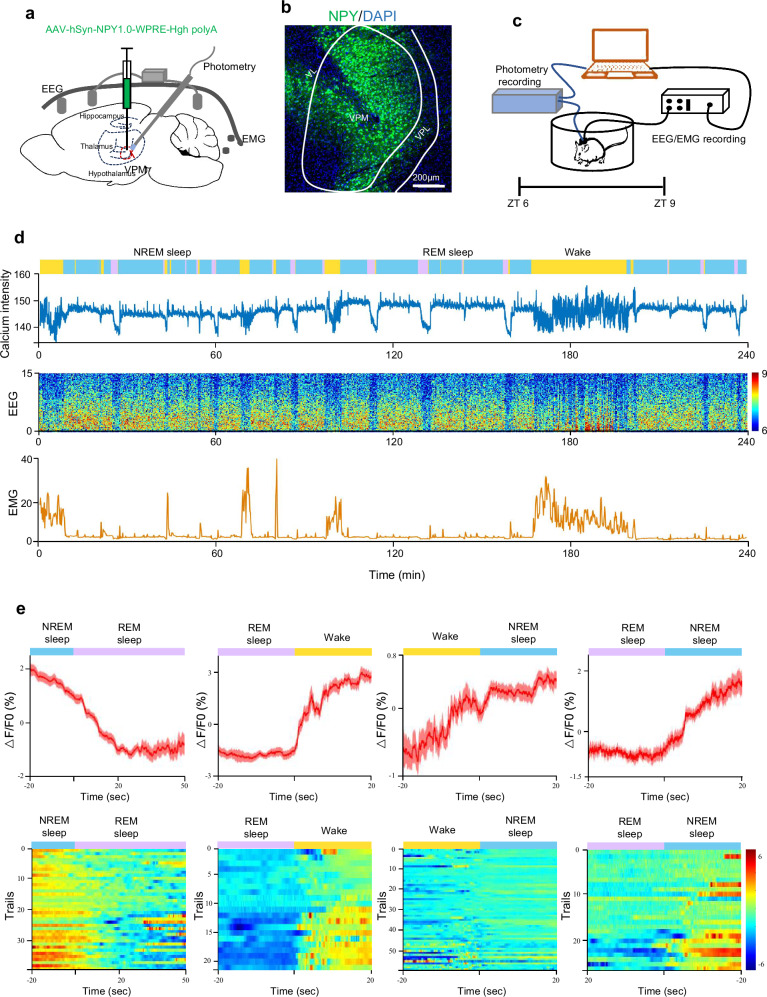
The dynamic pattern of NPY in the thalamus across wake-sleep cycle. **a** Schematic diagram of AAV-hSyn-GRAB_NPY_ virus injection and EEG/EMG electrode implantation. **b** Representative immunofluorescence image for GRAB_NPY_ expression in the thalamus. Scale bar, 200 µm. **c** Experimental design of synchronous EEG/EMG and photometry recordings from ZT 6 to ZT 9 (2 PM to 5 PM). ZT 0 indicates the start of the light period, and ZT 12 corresponds to the beginning of darkness. **d** Yellow, blue and purple bars showing wake, NREM sleep and REM sleep (Top). Middle, representative traces of NPY dynamics in the thalamus. Bottom, representative spectrogram from EEG and EMG. **e** Top, the temporal change of NPY at each brain state transition. Bottom, heatmaps showing NPY level at each brain state transition. *n* = 4 mice, recorded for 4 h from 2 PM to 5 PM. Data are presented as mean ± SEM.

Given that NPY is abundant in the central nervous system and involved in the regulation of circadian and sleep^39–42^, we speculated whether NPY is the key factor for the disrupted sleep behavior induced by *Mettl3* deficiency. To address this question, we first examined the level of NPY across the wake-sleep cycle. AAV-hSyn-G protein-coupled receptor (GPCR) activation-based (GRAB_NPY_) sensor for NPY was injected into the thalamus of WT mice (Fig. 7a, b). 4-h recording of extracellular NPY level during the light phase showed that the level of NPY exhibited an initial decline preceding each transition from NREM to REM sleep, followed by a significant surge upon the transitions from REM sleep to wake and from REM sleep to NREM sleep (Fig. 7c–e). These results suggested that NPY indeed displays a dynamic pattern in wake-sleep cycle.

**Fig. 8:**
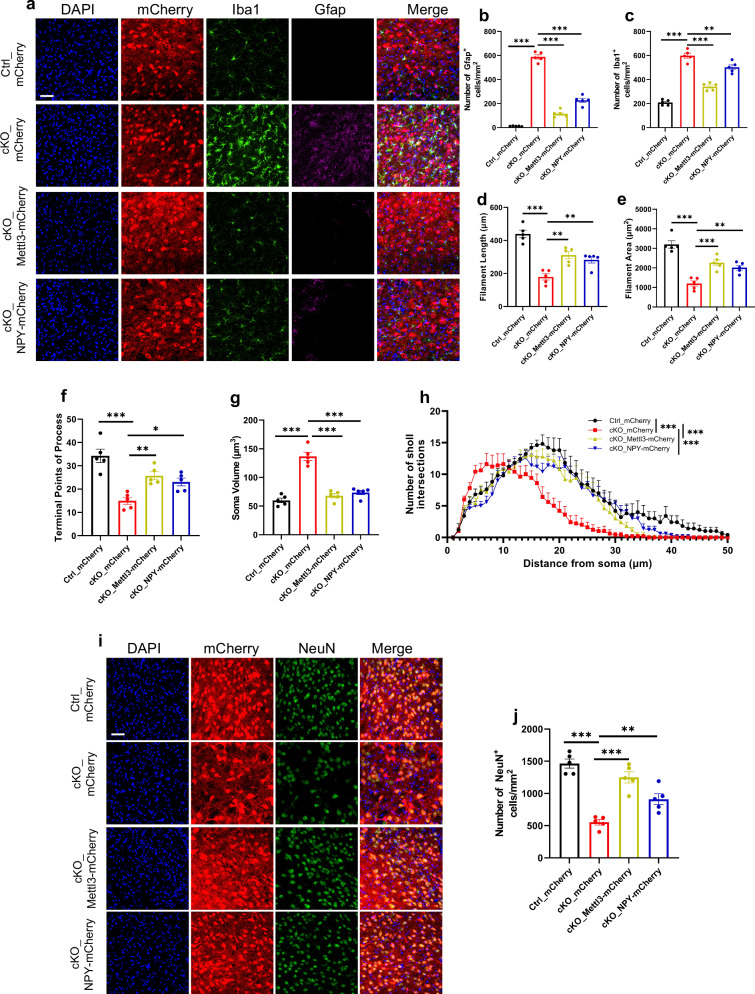
Ectopic expression of Mettl3 and NPY inhibits neuroinflammation and neuronal loss induced by *Mettl3* depletion. **a**–**c** Representative images of mCherry, Iba1 and GFAP immunofluorescence staining (**a**) and quantification of the numbers of GFAP^+^ astrocytes (**b**) and Iba1^+^ microglia (**c**) in the thalamus of 3-month-old Ctrl and cKO mice injected with AAV-mCherry, AAV-Mettl3-mCherry and AAV-NPY-mCherry. *n* = 5 mice for each group, and four sections were picked from each animal. Data are presented as mean ± SEM. One-way ANOVA followed by Tukey’s post hoc analysis, **P < 0.001, ***P < 0.0001. Scale bar, 50 µm. **d**–**h** Quantification of microglial filament length (**d**), filament area (**e**), terminal points of process (**f**), soma volume (**g**) and sholl analysis (**h**) of Iba1^+^ microglia in **a**. *n* = 5 mice for each group, and four sections were picked from each animal. Data are presented as mean ± SEM. One-way ANOVA followed by Tukey’s post hoc analysis for **d**, **e**, **g**, and **h**, **P < 0.001, ***P < 0.0001. Two-way ANOVA followed by Tukey’s post hoc analysis for **f**, *P < 0.05, **P < 0.001, ***P < 0.0001. **i**, **j** Representative images of mCherry and NeuN immunofluorescence staining (**i**) and quantification of the number of NeuN^+^ cells (**j**) in the thalamus of Ctrl and cKO mice. Mice were injected with AAV virus at the age of 2 months, and sacrificed 1 month later. *n* = 5 mice for each group, and four sections were picked from each animal. Data are presented as mean ± SEM. One-way ANOVA followed by Tukey’s post hoc analysis, **P < 0.001, ***P < 0.0001. Scale bar, 50 µm.

Next, we examined whether exogenous Mettl3 and NPY could alleviate neuropathology and restore disrupted sleep in cKO mice. AAV virus (AAV-hSyn-DIO-mCherry, AAV-hSyn-DIO-Mettl3, AAV-hSyn-DIO-NPY) was injected into bilateral VPM of adult (2-month-old) Ctrl and cKO mice, respectively (Supplementary Fig. S11a). 1.5 months post virus injection, western blot assay showed that AAV virus led to high expression of exogenous Mettl3 and NPY (Supplementary Fig. S11b–d). Immunofluorescence staining showed that ectopic expression of Mettl3 and NPY both remarkably reduced the numbers of Iba1^+^ microglia and GFAP^+^ astrocytes in cKO mice administered with AAV-Mettl3 and AAV-NPY compared to cKO mice administered with AAV-mCherry (Fig. 8a–c). Morphologic analysis showed that microglia displayed the increased length and area of filament, the increased intersection number and terminal points of the process and decreased soma volume in cKO mice administered with AAV-Mettl3 and AAV-NPY compared to cKO mice administered with AAV-mCherry (Fig. 8d–h). In addition, the number of NeuN^+^ neurons in the thalamus was significantly restored in AAV-Mettl3- and AAV-NPY-administered cKO mice compared to the Ctrl group (Fig. 8i, j). Collectively, these results suggested that ectopic NPY expression significantly inhibits the activation of microglia and astrocytes, and neuronal loss in the thalamus of cKO mice.

**Fig. 9:**
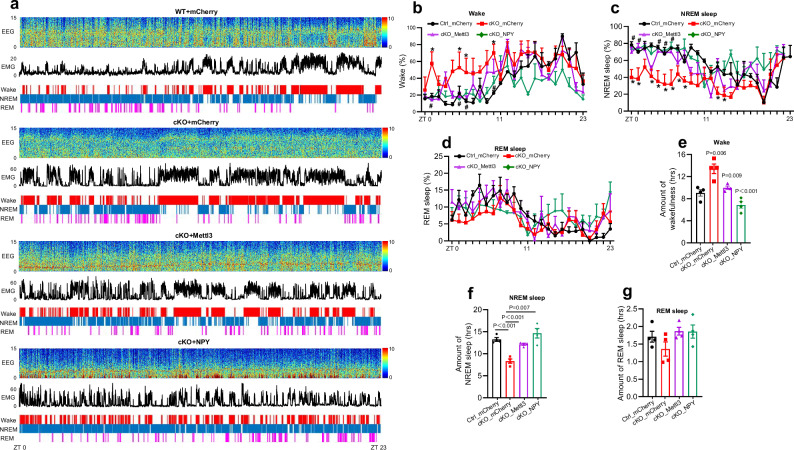
Ectopic expression of Mettl3 and NPY restores the disrupted sleep behavior of *Mettl3*-deficient mice. **a** Representative EEG/EMG recordings of Ctrl mice with AAV-mCherry injection (Ctrl_mCherry), and cKO mice with AAV-mCherry (cKO_mCherry), AAV-Mettl3-mCherry (cKO_Mettl3-mCherry) and AAV-NPY-mCherry (cKO_NPY-mCherry) injection spanning from ZT 0 to the following day at ZT 23. Top, representative spectrogram of EEG; middle, EMG; bottom, brain states annotated including wake, NREM sleep and REM sleep were shown. ZT 0 indicates the start of the light period, and ZT 12 corresponds to the beginning of darkness. **b**–**d** The percentage of time spent in the wake (**b**), NREM sleep (**c**) and REM sleep (**d**) in each hour throughout the 24-h recording. Data were analyzed using the two-way ANOVA with repeated measures. *P < 0.05, Ctrl_mCherry group vs cKO_mCherry group; #*P* < 0.05, Ctrl_mCherry group vs cKO_Mettl3-mCherry or cKO_NPY-mCherry. **e**–**g** The amount of time spent in the wake (**e**), NREM sleep (**f**) and REM sleep (**g**) across 24-h recording. *n* = 4 mice for each group. Data are presented as mean ± SEM. One-way ANOVA followed by Tukey’s post hoc analysis.

Next, we performed EEG/EMG recordings over a 24-h period 1 month post virus administration (Fig. 9a). We observed that ectopic expression of Mettl3 and NPY significantly reduced the proportion of wake state (*F* (3, 288) = 22.46, *P* < 0.0001) and increased the proportion of NREM sleep (*F* (3, 288) = 36.45, *P* < 0.0001) and REM sleep (*F* (3, 288) = 3.634, *P* = 0.0133) during the light-dark cycle relative to cKO mice administered with AAV-mCherry (Fig. 9b–d). In addition, the amount of wake state was significantly decreased (*F* (3, 12) = 19.60, *P* < 0.0001) and the amount of NREM sleep was remarkably increased (*F* (3, 12) = 18.43, *P* < 0.0001) in cKO mice administered with AAV-Mettl3 and AAV-NPY compared to cKO mice with AAV-mCherry, while the amount of REM sleep was not affected (*F* (3, 12) = 1.996, *P* = 0.1684) (Fig. 9e–g).

To verify the roles of NPY in regulating sleep behavior, we also adopted another strategy to express ectopic NPY (AAV-CaMKIIα-NPY-IRES-EGFP-3×FLAG-WPRE, AAV-EGFP-NPY) (Supplementary Fig. S11e). Immunofluorescence staining (Supplementary Fig. S11f, g) and western blot assay (Supplementary Fig. S11h, i) showed that AAV virus led to the efficient expression of NPY. Immunofluorescence staining and quantification results showed that ectopic expression of NPY remarkably reduced the number of Iba1^+^ microglia and intensity, increased the length and area of filament, increased the intersection number and terminal points, and decreased soma volume of Iba1^+^ microglia in thalamus of cKO mice relative to cKO mice administered with AAV-CaMKIIα-EGFP-3×FLAG-WPRE (AAV-EGFP) (Supplementary Fig. S12a–h). The number of GFAP^+^ cells was also significantly decreased in cKO mice administered with AAV-EGFP-NPY compared to cKO mice administered with AAV-EGFP (Supplementary Fig. S12i, j). qRT-PCR and western blot assay showed that ectopic NPY expression significantly reduced the levels of Iba1 and GFAP (Supplementary Fig. S12k–o), TNF-α and IL-1β (Supplementary Fig. S12p–t). In addition, the number of NeuN^+^ neurons was significantly restored in AAV-EGFP-NPY-administered cKO mice compared to AAV-EGFP-administered cKO mice (Supplementary Fig. S12u, v). Ectopic NPY expression also significantly reduced the activation of STAT3, but did not affect total STAT3 level (Supplementary Fig. S12w–y).

Next, we performed EEG/EMG recordings over a 24-h period (Supplementary Fig. S13a). Ectopic NPY expression driven by CaMKIIα promoter significantly reduced the proportion of wake state (*F* (2, 288) = 23.41, *P* < 0.0001) (Supplementary Fig. S13b), increased the proportion of NREM sleep (*F* (2, 288) = 25.26, *P* < 0.0001) (Supplementary Fig. S13c) as well as REM sleep distribution (*F* (2, 288) = 5.967, *P* < 0.0029) during the light-dark cycle (Supplementary Fig. S13d) relative to cKO mice administered with AAV-mCherry. Ectopic NPY expression significantly reduced wake amount (*F* (2, 12) = 7.599, *P* = 0.0074), increased NREM sleep amount (*F* (2, 12) = 11.38, *P* = 0.0017), and had no effects on REM sleep (*F* (2, 12) = 0.7736, *P* = 0.4831) (Supplementary Fig. S13e–g). Collectively, these results suggested that exogenous Mettl3 and NPY effectively restored the disrupted sleep behavior.

## Discussion

In the present study, we have revealed the critical function of Mettl3 in regulating sleep behavior via modulating *NPY*. Mettl3 is the core enzyme for catalyzing m^6^A modification. As the most prevalent mRNA modification, m^6^A directly or via interacting with other gene regulators regulates gene expression, including the export, stability, maturation and translation of mRNA, and is consequently involved in diverse biological processes^22,24,43–45^. In the neuronal system, m^6^A modification in mRNA intensively regulates astrogliosis, learning and memory, neurodevelopment and neurogenesis^21,22,24,44–51^. The multifaced features of m^6^A are not only indicated by its specific enrichment at distinct elements of mRNA but also suggested by its specific profile at distinct brain regions^52–54^. Our present study showed that *Mettl3* loss in the CamkIIα^+^ excitatory neurons reduced food intake and body and brain weights. Specifically, the activation of microglia and astrocytes, and neuronal loss were observed in the thalamus. Considering the widespread existence of CamkIIα^+^ excitatory neurons in the brain, it is plausible to speculate that neuronal loss in the thalamus is only one of the factors, and CamKIIα^+^ neurons in other brain regions could be involved in these deficits, even though other brain regions did not display significant neuronal loss.

Previous studies have shown that activated microglia trigger the activation of astrocytes^55,56^. The activated microglia and astrocytes are the main mediators of neuroinflammation and are involved in neurodegenerative and psychiatric diseases^57–61^. Our results showed that microglia activation is indeed prior to astrocyte activation and neuronal loss. In addition, activated microglia displayed phagocytic activity^55,56^, which could be the underlying mechanism for neuronal loss. All these deficits were induced by the specific deletion of *Mettl3* in neurons, suggesting the important function of Mettl3 in regulating the crosstalk between neurons and glia. Therefore, our study revealed novel roles of Mettl3-catalyzed m^6^A in regulating brain function.

NPY is present in multiple brain regions and plays pivotal neuromodulatory roles in food intake, neurogenesis, circadian rhythm and physical cognition^40–42,62–64^. NPY showed protective function under some pathological conditions, such as stress, ischemia, obesity and neuroinflammation^64–67^. Our results showed that NPY has the highest expression and m^6^A modification levels in the thalamus compared to other brain regions, such as the cortex and hippocampus. Consistently, *Mettl3* deficiency in excitatory neurons specifically reduced NPY levels in the thalamus, but not in other brain regions. Neuronal NPY regulates the homeostasis of microglia. Under conditions short of NPY, other mechanisms could be activated to maintain the homeostasis of microglia. When these mechanisms cannot neutralize the effects of NPY absence, microglia are activated and consequently induce neuronal loss. Therefore, our findings suggest the critical function of Mettl3 in regulating neuronal NPY and the homeostasis of microglia in the thalamus.

Sleep is a complex behavior, and multiple brain regions and neural circuits are involved in regulating sleep^1,7,8,68,69^. In our study, we found that CamkIIα^+^ excitatory neurons in the thalamus are critical for sleep. Neuronal *Mettl3* loss triggered severe neuroinflammation and, in turn, resulted in neuronal loss. Our results highlighted the function of Mettl3 in neurons of VPM, a thalamic nucleus related to sleep and arousal from anesthesia. Considering that the effect of *Mettl3* knockdown on sleep was weaker than that of *Mettl3* knockout in mice, and VPM is only part of the thalamus, the roles of neurons in other thalamic regions for sleep merit further study. In addition, our study showed that neurons in the thalamus were more sensitive to *Mettl3* loss. One potential mechanism is that Mettl3 had a higher binding affinity to *NPY* and conferred more m^6^A modification on *NPY* in the thalamus compared to the cortex and hippocampus. It is requisite to reveal the underlying mechanisms for the differential responses of neurons to *Mettl3* loss between brain regions.

Diverse mechanisms, including genetics, environmental signals, and metabolism, regulate sleep behavior^7,8,70^. Recent studies showed that DNA methylation age could be a marker for the early onset of REM sleep behavior disorder^17^. Histone deacetylases 4 and 5 regulate sleep amount via AMPK-related protein kinase SIK3^10,11^. In addition, many circadian clock gene transcripts are indeed modified by m^6^A, and *Mettl3* knockdown indeed affects the circadian period^71^, but the underlying mechanism and pathway remain unclear. Our results showed that *Mettl3* loss leads to the decreased expression of NPY in neurons of VPM, and induces sleep disorder, especially NREM sleep disorder. We found that NPY displayed a dynamic pattern in sleep. Consistently, patients with primary insomnia had lower NPY levels in the blood than normal controls^72^. Ectopic expression of Mettl3 and NPY reduced neuroinflammation and neuronal loss and rescued the disrupted sleep behavior, suggesting that neuronal NPY is an essential regulator of sleep and may serve as a therapeutic target for patients with sleep disorders.

## Materials and methods

### Animals

All mice used were in the C57BL/6 genetic background and were maintained in the animal center of Zhejiang University, Hangzhou, China, under 12-h light/12-h dark conditions with free access to food and water. *CaMKIIα-Cre* mice (Jax #005359) were purchased from the Jackson Laboratory and *Mettl3*^*flox/flox*^ mice were generated as previously described^73^. To generate mice with *Mettl3* cKO in excitatory neurons, *Mettl3*^*flox/flox*^ mice were crossed with *CaMKIIα-Cre* mice (Jax #005359) to generate heterozygous mice (*CaMKIIα-Cre;Mettl3*^*flox/+*^). Heterozygous mice were crossed with *Mettl3*^*flox/flox*^ mice to generate *CaMKIIα-Cre;Mettl3*^*flox/flox*^ mice (cKO) and littermate *Mettl3*^*flox/flox*^ mice (Ctrl). The genotypes of animals were determined by PCR with genomic DNA extracted from tail tissue. Only male mice were adopted for assays in the present study. All animal experiments were carried out following the protocols approved by the Zhejiang University Animal Care and Ethics Committee.

### Body weight and food intake monitoring

Each of Ctrl or cKO mice was fed separately under identical conditions. From postnatal 4 weeks, the body weight was monitored once 2 weeks for totally 18 weeks and the food intake was recorded after 12-h fasting every two weeks for totally 18 weeks.

### Stereotactic injection of the AAV virus

To deliver exogenous Mettl3 and NPY, AAV-hSyn-DIO-mCherry, AAV-hSyn-DIO-Mettl3-mCherry, AAV-hSyn-DIO-NPY-mCherry, AAV-CamkIIα-3×FLAG-EGFP and AAV-CamkIIα-NPY-3×FLAG-EGFP plasmids were constructed to ectopically express Mettl3 and NYP. For acute knockdown of *Mettl3*, scramble (CTCGCTTGGGCGAGAGTAA) and shRNA against *Mettl3* (TAAGCACACTGATGAATCTTT) were cloned into an AAV vector driven by the CamkIIα promoter, respectively. AAV virus was produced by OBiO (Shanghai, China). For the stereotactic injection, adult mice (postnatal 2 months) were anesthetized with tribromoethanol (0.2 mL/10 g body weight of a 1.2% solution) and installed on a stereotaxic instrument (DAVID KOPF Instruments, CA, USA). AAV was stereotaxically injected into the bilateral thalamus (0.2 μL virus in a titer of 10^8^ vg/mL per side) with a 10 μL syringe (#87930, Hamilton, USA) connected with a customed No.33 needle (#776206, Hamilton, USA). The following coordinates were used: 2.6 mm post-Bregma, 1.2 mm lateral to the midline and 2.75 mm below the dura. After the surgery, the mice were put back in their home cage and housed under standard conditions. 4 weeks post the AAV injection, mice were deeply anesthetized with 0.3% pentobarbital sodium (0.1 mL/10 g bodyweight, intraperitoneal) and transcardially perfused with cold phosphate buffer saline (PBS) followed by cold 4% paraformaldehyde (PFA). All mice were examined for the location and efficiency of AAV injection after behavioral experiments.

### Sleep pattern monitoring

As described previously^74,75^, four stainless steel wire leads implanted and fixed bilaterally onto the frontal and partial cortices and two Teflon-coated stainless steel wires placed bilaterally within the dorsal neck musculature under surgical anesthesia were used as the electrodes for EEG and EMG recordings, respectively.

After mice recovered for 7 days, EEG/EMG signals were recorded using a Medusa small animal electrophysiology recording system (Medusa, Bio-Signal Technologies, Nanjing, Jiangsu, China) with a sampling frequency of 1000 Hz from ZT 0 to the following day at ZT 23. The data were analyzed with the customed MATLAB software^74–77^. Briefly, brain states were scored at 10 s epochs, and slow-wave sleep (NREM sleep) was identified by heightened delta (1–4 Hz) power in the EEG alongside subdued EMG activity. REM sleep was characterized by an increase in theta (6–10 Hz) power in the EEG accompanied by minimal EMG signals. Wake was discerned by EEG patterns displaying low amplitude and high-frequency activity, coupled with tonic EMG activity.

### Fiber photometry

Adult (8-week-old) C57BL/6 J mice were anesthetized using tribromoethanol (0.2 mL/10 g body weight of a 1.2% solution). Following anesthesia induction, the mice were carefully positioned on the stereotaxic apparatus. AAV2/9-hSyn-NPY 1.0 virus (200 nL, Brain Case, Shenzhen, China) was then injected into the VPM (AP –1.94 mm, ML + 1.5 mm, DV –3.5 mm) at a rate of 20 µL/min to monitor NPY levels during the sleep-wake cycle. The AAV2/9-hSyn-NPY 1.0 virus (200 nL) was injected into the VPM region to monitor NPY levels during sleep-wake cycle. Following virus injections, an optical fiber housed within a ceramic ferrule was meticulously inserted into the VPM region. Subsequently, it was securely affixed alongside the EEG/EMG electrode plug. The NPY signals and sleep transitions were synchronously recorded from ZT 6 to ZT 9. NPY signals were recorded using a commercial photometry system (Thinker Tech Nanjing Biotech Co., Ltd., China). NPY-dependent fluorescence signals were obtained by the VPM neurons expressing NPY sensor with a 470 nm laser. Photometry data were imported into MATLAB R2020b MAT files for subsequent analysis as described previously^74^. The data underwent smoothing using a moving average filter and segmentation based on behavioral events. Fluorescence change values (Δ*F*/*F*) were calculated using the formula (*F* – *F*_0_)/*F*_0_, where *F*_0_ represents the baseline fluorescence signal averaged over a 1.5-s control time window. These Δ*F*/*F* values were visualized through spectrograms or average plots, with shaded areas indicating the standard error of the mean (SEM).

### Isolation and culture of primary astrocytes

The isolation and culture of astrocytes from postnatal day 1 (P1) mouse pups were prepared as described previously^78^. P1 mouse pups were sacrificed and the cortical tissue was taken out. The brain tissue was washed with PBS and cut into pieces, and treated with 0.25% trypsin-EDTA at 37 °C for 10–15 min. Then 40 μL deoxyribonuclease I (50,000 U/mL) was added and digested for 5 min. Digestion was terminated with DMEM medium containing 10% FBS. After centrifugation at 1600 rpm for 8 min, the supernatant was discarded, and the cell precipitate was resuspended in a DMEM medium containing 10% FBS, 2 mM l-glutamine and 1% antibiotics. Then the cells were seeded on the cell culture plate coated with PDL (5 μg/mL). The cells were cultured at 37 °C in a 5% CO_2_ incubator. After 24 h, the medium was completely replaced, and the full medium was replaced every 2 days. When the cell fusion reached ~95%, the sample was purified by shaking at 37 °C for 16–18 h (260 rpm). Astrocytes were passaged once with 0.25% trypsin-EDTA for RNA extraction.

### Isolation and culture of primary neurons

The isolation and culture of primary neurons were prepared as described previously^79^. Fetal cortical tissue was isolated from the mouse fetal brains at embryonic day 17 (E17) and washed with ice-cold PBS, digested with 0.25% trypsin at 37 °C for ~30 min. After the digestion was terminated, the cells were centrifuged and resuspended, and ~150,000 cortical neurons were seeded on a cell climbing sheet coated with poly d-lysine (5 μg/mL, Sigma, P0899-10). The cells were grown in plate medium containing MEM (Gibco, 11095-080), 10% FBS (Gibco, 10091-148), 1% l-glutamine (Gibco, 5030-149), 1% sodium pyruvate (Gibco, 11360-070), 0.45% d-glucose (Amresco, 0188, Radnor, PA, USA). After 4 h, the medium was replaced with neurobasal medium (Gibco, 21103-049), supplemented with 2% B27 (Gibco, 17504-044), 0.25% l-glutamine (Gibco, 25030-149), 0.125% Glutamax (Thermo, 35050061). 1/2 of the liquid medium was replaced every 3 days. RNA extraction was performed after ~14 days of culture.

### BV2 cell culture

Mouse microglia cell line BV2 (Corning, Cat# 10-013-CV, USA) was cultured in high-glucose DMEM medium containing 10% FBS, 10 mM HEPES (Gibco, Cat# 15630080, USA), 1% penicillin and streptomycin (BBI, Cat# E607018, China). The cells were passaged by digesting with 0.25% trypsin-EDTA at 37 °C for 2–3 min, and then the digestion was terminated using the culture medium. The cells were passaged at a ratio of 1:3 and cultured in an incubator at 37 °C, 5% CO_2_.

### Immunofluorescence staining

Mice were deeply anesthetized with 0.3% pentobarbital sodium, and transcardially perfused with cold PBS followed by cold 4% PFA. Brain samples were harvested, and post-fixed with 4% PFA overnight and completely dehydrated with 30% sucrose at 4 °C. Brain samples were embedded with OCT (4583, SAKURA) and 30-μm thickness sections were prepared with a cryostat (CM1950, Leica).

For the immunofluorescence staining assay, brain sections were washed with PBS for 30 min, and then were blocked with PBS containing 3% goat serum and 0.1% Triton X-100 for 1 h at room temperature. Sections were incubated with primary antibodies at 4 °C overnight. The following primary antibodies were used: mouse anti-CamkIIα (50049, Cell Signaling Technology, 1:500), rabbit anti-Mettl3 (Ab195352, Abcam, 1:500), rabbit anti-Iba1(Ab178847, Abcam, 1:500), mouse anti-GFAP (3670S, Cell Signaling Technology, 1:500), mouse anti-NeuN (MAB377, Millipore, 1:500), rabbit anti-S100β (Z0311, Dako, 1:500), rabbit anti-PSD95 (3450, Cell Signaling Technology, 1:500), rat anti-CD68 (NBP2-33337, Novus Biologicals, 1:500), mouse anti-NPY (bs-0071R, Bioss, 1:500), rabbit anti-NPY1r (bs-1070R, Bioss, 1:200). On the second day, samples were washed with PBS and incubated with fluorescence-conjugated secondary Alexa antibodies and DAPI (4′,6-diamidino-2-phenylindole) (D8417, Sigma) for nucleus staining at room temperature for 1 h. After being washed with PBS three times, sections were mounted on glass slides with a mounting medium. Images were captured with a confocal microscope (Olympus, FV3000) and analyzed with ImageJ software. The secondary antibodies used were AlexaFluor 488 goat anti-rabbit (A11008, Thermo Fisher Scientific, 1:500), AlexaFluor 488 goat anti-mouse (A11001, Thermo Fisher Scientific, 1:500), AlexaFluor 568 goat anti-mouse (A11004, Thermo Fisher Scientific, 1:500), AlexaFluor 568 goat anti-rabbit (A11036, Thermo Fisher Scientific, 1:500) and AlexaFluor 568 goat anti-rat (A11077, Thermo Fisher Scientific, 1:500).

### Sholl analysis

3D morphological analysis of Iba1^+^ microglia and GFAP^+^ astrocytes was performed with Imaris software (v9.3.1). The ‘Filament’ module and the ‘Autodepth’ algorithm were applied to reconstruct the framework and restore processes of microglia and astrocytes completely and precisely. To outline the cell’s framework, the cell body was set as a central point and process terminals were used as the endpoints. 1 μm was set as the spacing of Sholl analysis to quantify the extent of process branching. The total length and area of the process, as well as the number of endpoints and soma volume, were also indicated.

### Total RNA isolation and real-time PCR

Total RNA of brain tissues was extracted with TRIzol reagent following the manufacturer’s protocol (15596018, Invitrogen). The concentration of RNA was measured by NanoDrop spectrophotometer 2000 (Thermo Fisher Scientific) and 1 μg of total RNA was used for reverse transcription. qRT-PCR was performed using SYBR Green qPCR mix (Q711, Vazyme) in triplicate, and measured by Applied Biosystems Viiia 7. Gapdh was used as the internal control. The final results were calculated by ΔΔCt method. The primers used for qRT-PCR are shown in Supplementary Table S1.

### RNA-seq analysis

Total RNAs of thalamic tissues from 2- and 4-month-old Ctrl and cKO mice were extracted for RNA-seq, respectively. All samples used for the cDNA library were evaluated using NanoDrop 2000 (Thermo Fisher Scientific), and the RNA integrity value (RIN) was measured using the RNA Nano 6000 Assay Kit of the Bioanalyzer 2100 system (Agilent Technologies Inc.). A total amount of 2 μg RNA was used for each RNA sample preparation. Sequencing libraries were generated according to the protocol of NEBNext Ultra^TM^ RNA Library Prep Kit for Illumina (NEB). The library was sequenced with the Illumina Hiseq platform (Illumina NovaSeq 6000).

Raw reads of RNA-seq data were processed, and clean reads were gained from raw reads by removing low-quality reads and reads containing adapter or ploy-N. Then the remaining clean reads were mapped to the Mus musculus genome (mm10) using Hisat2 v2.2.1. FeatureCounts v2.0.1 was used to count the read numbers mapped to each gene to quantify the gene expression level. Fragments per kilobase of transcript per million mapped reads (FPKM) of genes were calculated based on the length of the genes and the gene mapping of read counts. The analysis of differential expression was performed using the edgeR package (3.32.1). An adjusted *P*-value of 0.05 and an absolute fold change of 1.5 were set as the threshold for significantly differentially expressed genes.

### GO analysis

GO analysis was performed using the DAVID database, as described previously^24^. Each enriched GO function term was represented by a node, and the node size was proportional to the number of genes in its corresponding function term in the enrichment maps. Similar GO functions were categorized as one cluster. The function term and the number of genes in each cluster were labeled.

### Western blot assay

Cells and brain tissues, including the thalamus, hippocampus and retrosplenial cortex were dissected on ice and quickly frozen with liquid nitrogen before total protein extraction. Tissues were triturated with cold RIPA Lysis Buffer on ice and transferred to a microfuge tube followed by centrifugation at 4 °C for 30 min at 12,000 rpm. Cell pellets were collected and the total proteins were lysed with cold RIPA Lysis Buffer on ice and then centrifuged at 4 °C for 30 min at 12,000 rpm. Supernatants were collected and quantified by BCA protein assay.

Proteins were subjected to SDS-PAGE at different concentrations of separating gels according to molecular weights of target proteins and then transferred onto nitrocellulose membranes. Membranes were blocked with 5% non-fat milk or 5% BSA (MB4219, Meilunbio) in TBST at room temperature for 1 h and incubated with primary antibodies at 4 °C overnight. The following primary antibodies were used: rabbit anti-Mettl3 (Ab195352, Abcam), rabbit anti-Iba1 (Ab178847, Abcam), mouse anti-GFAP (3670S, Cell Signaling Technology), rabbit anti-TNF-a (11948S, Cell Signaling Technology), mouse anti-IL-1b (Ab9722, Abcam), rabbit anti-PSD95 (3450, Cell Signaling Technology), mouse anti-VGluT1 (MAB5502, Millipore), rat anti-CD68 (NBP2-33337, Novus Biologicals), mouse anti-NPY (bs-0071R, Bioss), rabbit anti-Cleaved caspase3 (9664S, Cell Signaling Technology), rabbit anti-pStat3 (9145S, Cell Signaling Technology), mouse anti-Stat3 (9139S, Cell Signaling Technology) and mouse anti-Gapdh (AC033, Abclonal).

After being washed with TBST for three times, membranes were incubated with HRP-labeled secondary antibodies at room temperature for 1 h. Membranes were washed three times with TBST and incubated with UltraSignal ECL (4AW001, 4A Biotech). The targeted bands were incubated with UltraSignal ECL (4AW001, 4A Biotech), and detected by Tanon Detection system (Tanon 5200). The intensity was analyzed with Adobe Photoshop software.

### m^6^A dot blot assay

Firstly, the total RNA sample extracted from brain tissue (400 ng) was mixed with 37% formaldehyde and 3-morpholine propanesulfonic acid (MOPS) solution, treated at 65 °C for 10 min, and then cooled on ice. RNA sample was directly spotted on Hybond N^+^ membrane (NP1096, GE Healthcare), and air-dried at 65 °C for 45 min. After being crosslinked for 5 min by ultraviolet, the membrane was blocked with 5% skimmed milk powder in TBS for 1 h, and 2 µg anti-m^6^A antibody (A22411, ABclonal) was added to incubate at 4 °C overnight. On the second day, the membrane was rinsed with TBS 3 times and incubated with HRP-labeled anti-rabbit IgG secondary antibody at room temperature for 2 h. The signal was detected with the Tanon Detection system (Tanon 5200), and methylene blue staining was performed to verify equal loading of samples. Photoshop software was used to quantify the signal density.

### RIP

RIP experiment was carried out with several modifications^80^. Briefly, fresh thalamic tissues were homogenized on ice with RIP buffer (50 mM Tris-HCl, pH 7.4, 1 mM MgCl_2_, 1 mM DTT, 150 mM NaCl, 1% NP-40, RNase inhibitor and protease inhibitor cocktail) for 30 min. The homogenate was centrifuged at 15,000× *g* for 30 min, the supernatant was collected, and the precipitate was discarded. For the RIP reaction of each sample, 10% of the lysate supernatant was removed as 10% input. The remaining supernatant was incubated with Mettl3 antibody-binding protein A/G magnetic beads at 4 °C overnight. On the next day, the immunoprecipitation complex was washed three times with high-salt buffer (300 mM NaCl, 50 mM Tris-HCl, pH 7.4) and low-salt buffer (150 mM NaCl, 50 mM Tris-HCl, pH 7.4), respectively. RNA was eluted from the magnetic beads with elution buffer (containing 1% SDS and 20 mg/mL Proteinase K) and extracted. 500 ng of Mettl3-conjugated mRNAs were reverse transcribed and quantified by qPCR. 10% input was normalized to calculate the corresponding Mettl3-enriched RNA level in each sample.

### m^6^A MeRIP-qPCR

The total RNA of brain tissues was extracted with Trizol reagent, and purified with RNA mRNA purification kit (61006, Thermo Fisher Scientific). The concentration of mRNA was quantified using NanoDrop One, and 10% of the total mRNA was taken out as input. 2–3 μg of anti-m^6^A antibody (A22411, ABclonal) and rabbit IgG were incubated with prewashed Protein A Magnetic Beads (10002D, Thermo Fisher Scientific) at 4 °C for 4–6 h, respectively. Beads were washed with IPP buffer (150 mM NaCl, 0.1% NP-40, 10 mM Tris-HCl, pH 7.4), and m^6^A antibody-conjugated beads were mixed with purified poly (A)-mRNA and incubated at 4 °C overnight. After the sample was taken out, m^6^A-conjugated mRNA was eluted with 0.5 mg/mL *N*^6^-methyladenosine (Cat# P3732; Berry & Associates, MI) at 4 °C overnight, and m^6^A-conjugated mRNA was precipitated with TRIzol reagent and absolute ethanol. m^6^A-conjugated mRNA was reverse transcribed and quantified by qPCR. 10% input was normalized to calculate the corresponding m^6^A enrichment level in each brain sample.

### Prediction of m^6^A modification site

*NPY* mRNA sequence was obtained from the National Center for Biotechnology Information (NCBI, https://www.ncbi.nlm.nih.gov). m^6^A modification site of *NPY* mRNA was predicted with the Sequence-based RNA adenosine methylation site predictor (SRAMP, http://www.cuilab.cn/sramp)^81^. The classical m^6^A modification motif (GGACU) was predicted to be located on exon 2 of *NPY*. Primers were designed before and after the m^6^A motif for RIP-qPCR and MeRIP-qPCR (Supplementary Table S1).

### Statistical analysis

All data are expressed as mean ± SEM using GraphPad Prism (GraphPad Software, CA, USA). The differences were assessed using a two-tailed unpaired Student’s *t*-test between two groups or one-way and two-way ANOVA analyses followed by post hoc Tukey’s test for comparison as appropriate. A statistical significance was considered when a *P* value is < 0.05. The details for the replicates of each experiment are indicated in the figure legends.

## Supplementary information

Supplementary Figures

Supplementary Table S1

Supplementary Table S2

Supplementary Table S3

## Data Availability

The RNA-seq data reported in this study have been deposited at the NCBI GEO with accession number GSE262033.
