# Supplementary material for: Mettl3-m6A-NPY axis governing neuron–microglia interaction regulates sleep amount of mice
Source: Cell Discov. 2025 Feb 4;11:10. doi: 10.1038/s41421-024-00756-y (PMC11794856; doi:10.1038/s41421-024-00756-y)
Supplement: Supplementary file 1 — Supplementary Figures [file 41421_2024_756_MOESM1_ESM.pdf]

Mettl3-m^{6}A-Npy axis governing neuron-microglia interaction regulates sleep amount of mice

Qihang Sun[1,2,*], Jinpiao Zhu[1,3,*,#], Xingsen Zhao[4,*], Xiaoli Huang[1], Wenzheng Qu[1], Xia Tang[1], Daqing Ma[3,5,#], Qiang Shu[1,#], Xuekun Li[1,2,6,#]

Supplementary Fig. 1

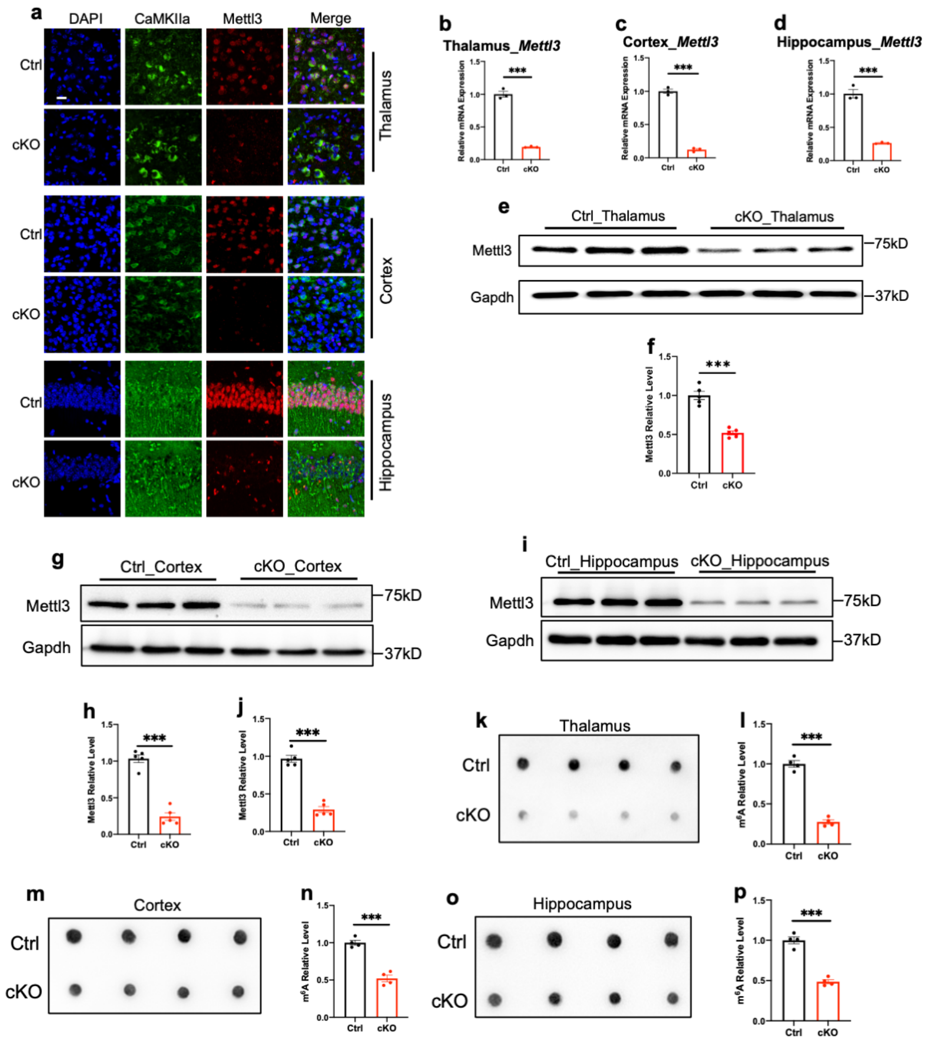

continued Supplementary Fig. 1

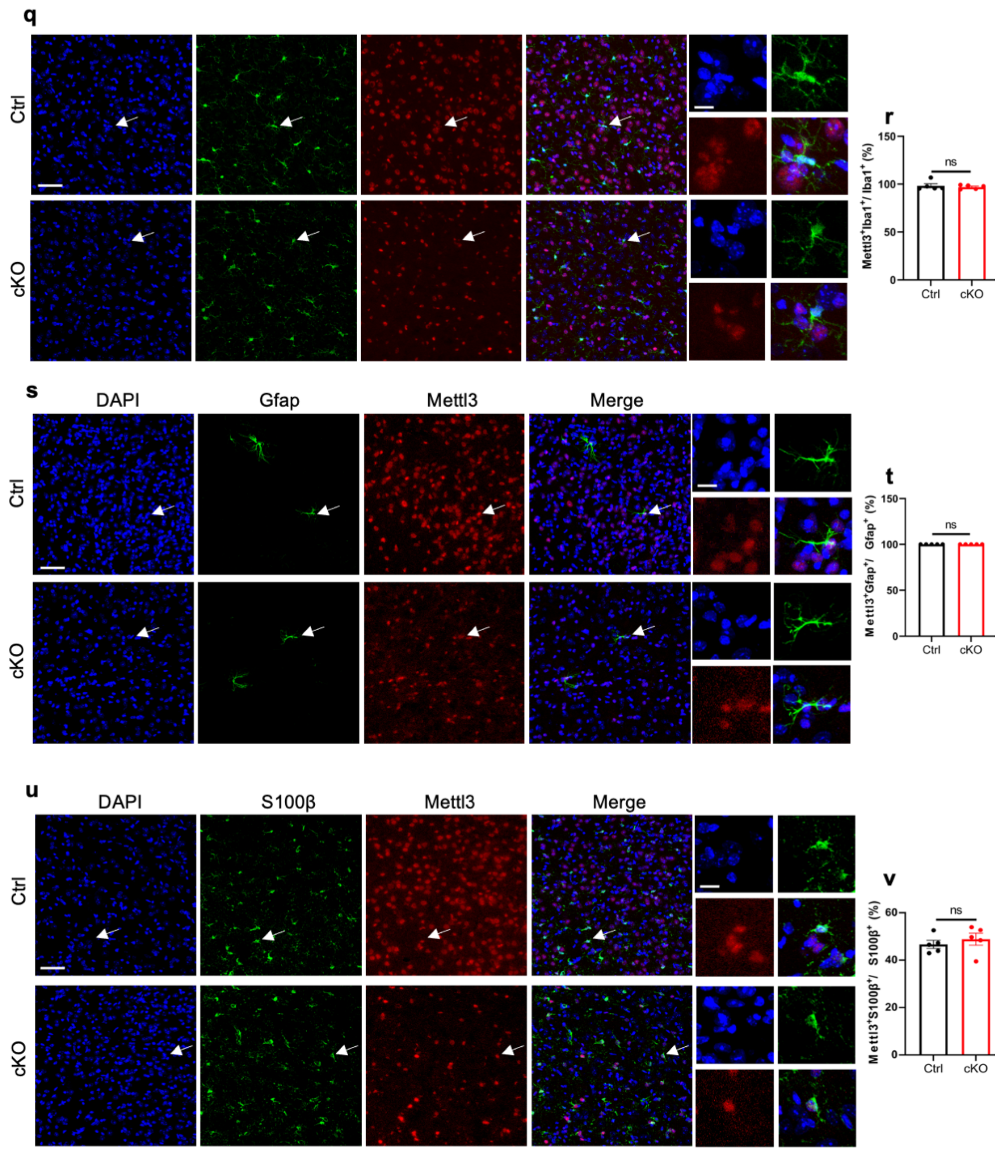

Supplementary Fig. S1. ***Mettl3*** **deficiency reduces the levels of Mettl3 and m^{6}A in mouse brain.**

a. Representative images of CaMKIIα and Mettl3 immunofluorescence staining in thalamus, hippocampus and retrosplenial cortex of 4-month-old Ctrl and cKO mice, respectively. Scale bar, 20 μm.

b-d. qRT-PCR assay of *Mettl3* mRNA expression in the thalamus (**b**), retrosplenial cortex (**c**) and Hippocampus (**d**) of 4-month-old mice. n=3 independent experiments for each group. Data are presented as mean ± SEM. Unpaired Student's t test, *p<0.05, **p<0.001, ***p<0.0001. The primers used can be found in Supplementary Table S1.

e, f. WB assay (**e**) and quantification results of Mettl3 (**f**) level in the thalamus of 4-month-old Ctrl and cKO mice. n=5 mice for each group. Data are presented as mean ± SEM. Unpaired Student's t test, *p<0.05, **p<0.001, ***p<0.0001.

g, **h.** WB assay (**g**) and quantification results of Mettl3 (**h**) level in retrosplenial cortex of 4-month-old Ctrl and cKO mice. n=5 mice for each group. Data are presented as mean ± SEM. Unpaired Student's t test, *p<0.05, **p<0.001, ***p<0.0001.

i, j. WB assay (**i**) and quantification results of Mettl3 (**j**) level in hippocampus of 4-month-old Ctrl and cKO mice. n=5 mice for each group. Data are presented as mean ± SEM. Unpaired Student's t test, *p<0.05, **p<0.001, ***p<0.0001.

k, l. Dot-blot assay (**k**) and quantification (**l**) showed that m^6A level was significantly decreased in thalamus of cKO mice compared to Ctrl mice at the age of 4-month. n=4 independent experiments for each group. Data are presented as mean ± SEM. Unpaired Student's t test, *p<0.05, **p<0.001, ***p<0.0001.

m, n. Dot-blot assay (**m**) and quantification (**n**) showed that m^6A level was significantly decreased in retrosplenial cortex of cKO mice compared to Ctrl mice at the age of 4-month. n=4 independent experiments for each group. Data are presented as mean ± SEM. Unpaired Student's t test, *p<0.05, **p<0.001, ***p<0.0001.

o, p. Dot-blot assay (**o**) and quantification (**p**) showed that m^6A level was significantly decreased in the hippocampus of cKO mice compared to Ctrl mice at the age of 4-month. n=4 independent experiments for each group. Data are presented as mean ± SEM. Unpaired Student's t test, *p<0.05, **p<0.001, ***p<0.0001.

q, r. Representative images of immunofluorescence staining (**q**) and quantification of the percentage of $Mettl3^+Iba1^+/Iba1^+$ (**r**) in thalamus of 2-month-old Ctrl and cKO mice. n=5 mice for each group, and 4 sections were picked up from each animal. Data are presented as mean ± SEM. Unpaired Student's t test, *p<0.05, **p<0.001, ***p<0.0001. Scale bars, 20 μm for left panels and 10 μm for right panels with higher magnification.

s, t. Representative images of immunofluorescence staining (**s**) and quantification of the percentage of $Mettl3^+Gfap^+/Gfap^+$ (**t**) in thalamus of 2-month-old Ctrl and cKO mice. n=5 mice for each group, and 4 sections were picked up from each animal. Data are presented as mean ± SEM. Unpaired Student's t test, *p<0.05, **p<0.001, ***p<0.0001. Scale bars, 20 μm for left panels and 10 μm for right panels with higher magnification.

u, v. Representative images of immunofluorescence staining (**u**) and quantification of the percentage of $Mettl3^+S100\beta^+/S100\beta^+$ (**v**) in thalamus of 2-month-old Ctrl and cKO mice. n=5 mice for each group, and 4 sections were picked up from each animal. Data are presented as mean ± SEM. Unpaired Student's t test, *p<0.05, **p<0.001, ***p<0.0001. Scale bars, 50 μm for left panels and 10 μm for right panels with higher magnification.

Supplementary Fig. 2

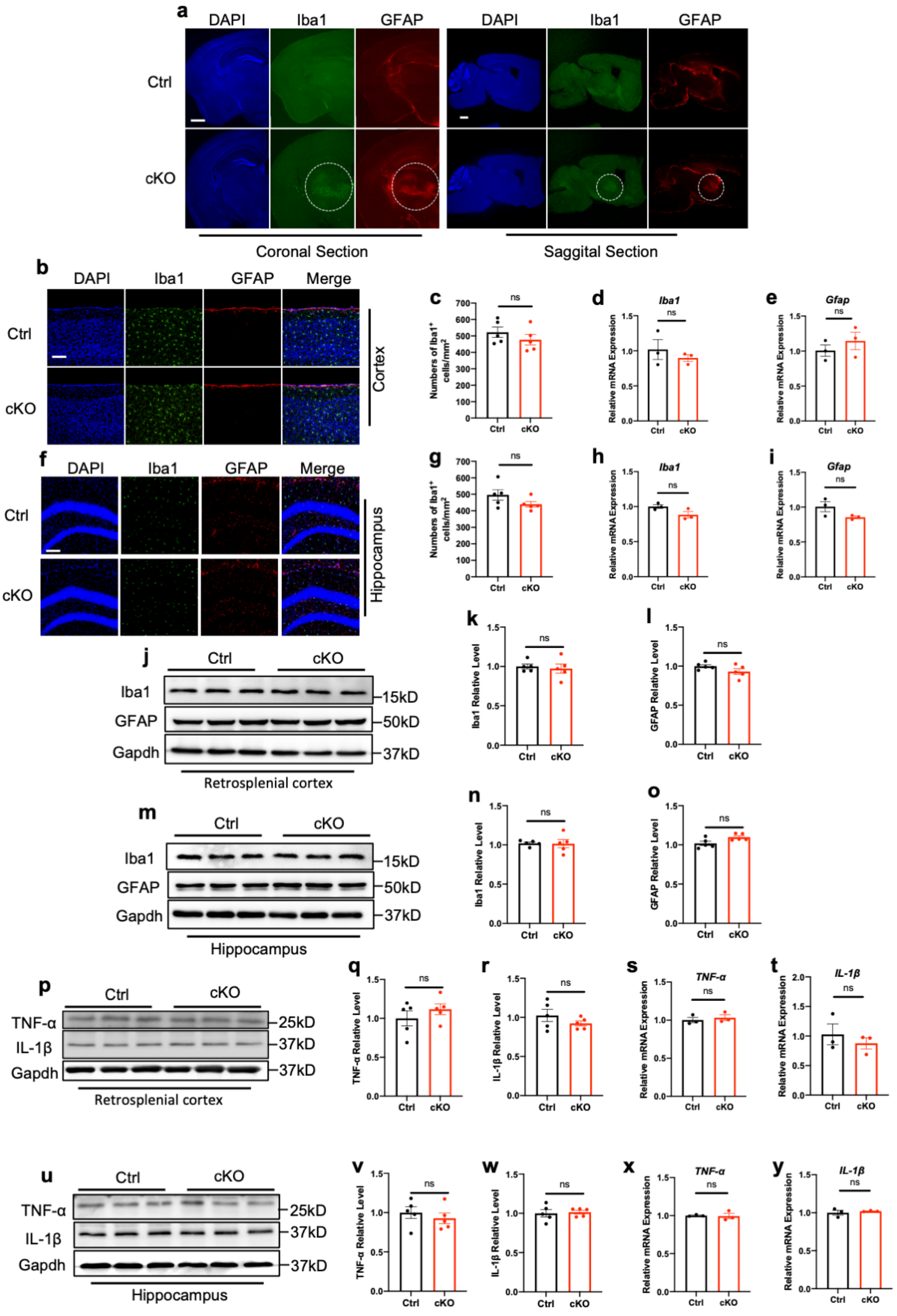

Supplementary Fig. S2. Neuronal *Mettl3* loss specifically induce neuroinflammation in the thalamus, but not in cortex and hippocampus.

a. Representative images of Iba1 and GFAP immunofluorescence staining in brain regions of 4-month-old Ctrl and cKO mice. Dashed lines circled the area of thalamus. Left, coronal section; right, sagittal section. Scale bar, 1 mm.

b, b. Representative images of Iba1 and GFAP immunofluorescence staining (**b**) and quantification of the number of Iba1$^+$ cells (**c**) in retrosplenial cortex of 4-month-old Ctrl and cKO mice. n=5 mice for each group, and 3 sections were picked up from each animal. Data are presented as mean ± SEM. Unpaired Student's t test, *p<0.05, **p<0.001, ***p<0.0001. Scale bar, 100 μm.

d, e. qRT-PCR assay of *Iba1* (**d**) and *Gfap* (**e**) mRNA expression in retrosplenial cortex of 4-month-old mice. n=3 independent experiments for each group. Data are presented as mean ± SEM. Unpaired Student's t test, *p<0.05, **p<0.001, ***p<0.0001.

f, g. Representative images of Iba1 and GFAP (**f**) immunofluorescence staining and quantification of the number of Iba1$^+$ cells (**g**) in hippocampus of 4-month-old Ctrl and cKO mice. n=5 mice for each group, and 3 sections were picked up from each animal. Data are presented as mean ± SEM. Unpaired Student's t test, *p<0.05, **p<0.001, ***p<0.0001. Scale bar, 100 μm.

h, i. qRT-PCR assay of *Iba1* (**h**) and *Gfap* (**i**) mRNA expression in hippocampus of 4-month-old mice. n=3 independent experiments for each group. Data are presented as mean ± SEM. Unpaired Student's t test, *p<0.05, **p<0.001, ***p<0.0001.

j-l. WB assay (**j**) and quantification results of Iba1 (**k**) and GFAP (**l**) in retrosplenial cortex of 4-month-old Ctrl and cKO mice, respectively. n=5 mice for each group. Data

are presented as mean ± SEM. Unpaired Student's t test, *p<0.05, **p<0.001, ***p<0.0001.

m-o. WB assay (**m**) and quantification results of Iba1 (**n**) and GFAP (**o**) in hippocampus of 4-month-old Ctrl and cKO mice, respectively. n=5 mice for each group. Data are presented as mean ± SEM. Unpaired Student's t test, *p<0.05, **p<0.001, ***p<0.0001.

p-r. WB assay (**p**) and quantification results of TNF-α (**q**) and IL-1β (**r**) in retrosplenial cortex of 4-month-old Ctrl and cKO mice, respectively. n=5 mice for each group. Data are presented as mean ± SEM. Unpaired Student's t test, *p<0.05, **p<0.001, ***p<0.0001.

s, t. qRT-PCR assay of *TNF-α* (**s**) and *IL-1β* (**t**) mRNA in cortex of 4-month-old mice. n=3 independent experiments for each group. Data are presented as mean ± SEM. Unpaired Student's t test, *p<0.05, **p<0.001, ***p<0.0001.

u-w. WB assay (**u**) and quantification results of TNF-α (**v**) and IL-1β (**w**) in hippocampus of 4-month-old Ctrl and cKO mice, respectively. n=5 mice for each group. Data are presented as mean ± SEM. Unpaired Student's t test, *p<0.05, **p<0.001, ***p<0.0001.

x, y. qRT-PCR assay of TNF-α (**x**) and IL-1β (**y**) mRNA expression in hippocampus of 4-month-old Ctrl and cKO mice. n=3 independent experiments for each group. Data are presented as mean ± SEM. Unpaired Student's t test, *p<0.05, **p<0.001, ***p<0.0001.

Supplementary Fig. 3

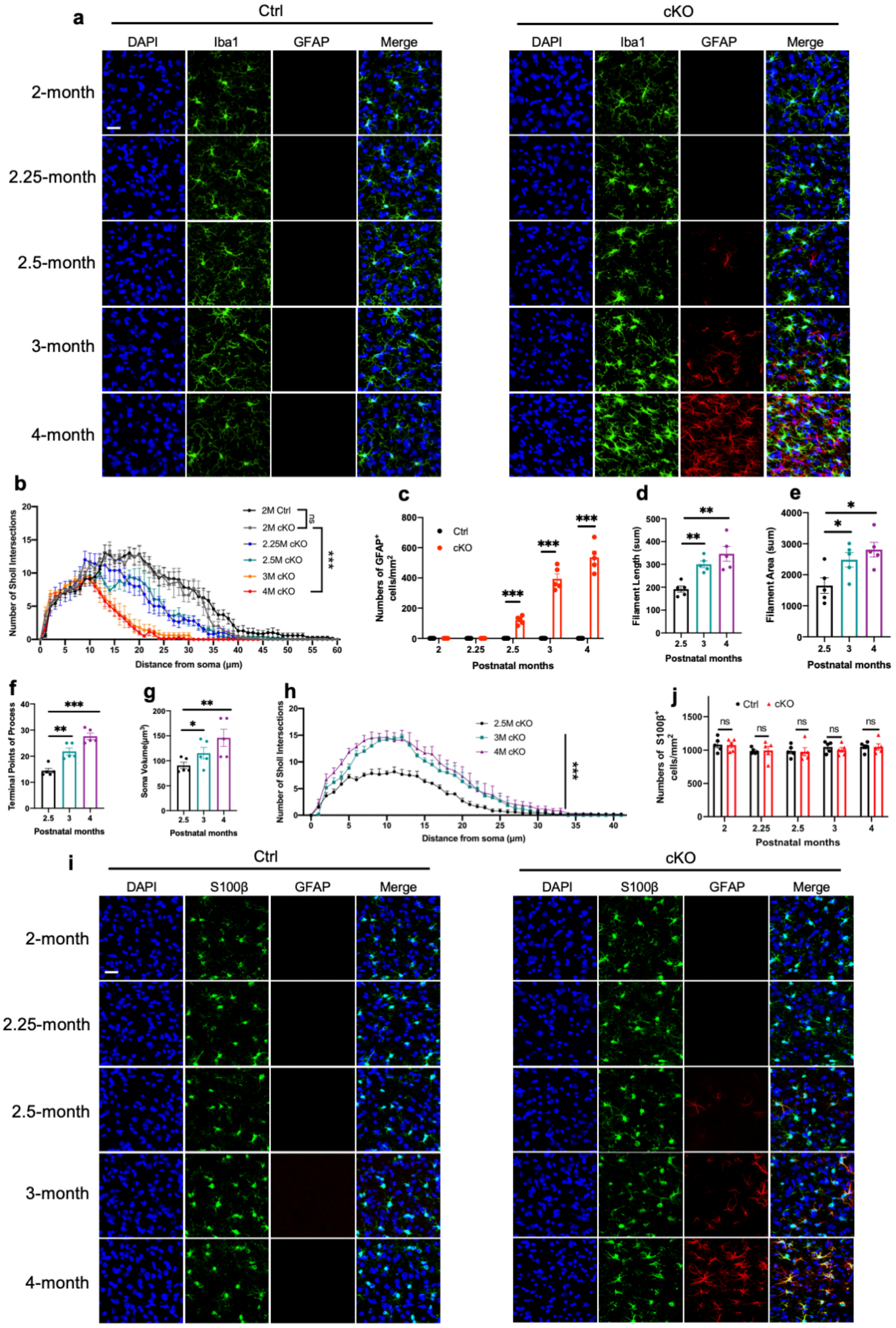

Supplementary Fig. S3. The activation of microglia induced by *Mettl3* loss is prior to the activation of astrocytes.

a. Representative images for Iba1 and GFAP immunofluorescence staining in thalamus of Ctrl and cKO mice at different ages. Scale bar, 20 μm.

b. Sholl analysis of Iba1^{+} microglia in thalamus of Ctrl and cKO mice at different ages. n=5 mice for each group, and 3 sections from the anterior, middle and posterior of thalamus were picked up for each animal. Data are presented as mean ± SEM. Two-way ANOVA analysis followed by Tukey's post hoc analysis, *p<0.05, **p<0.001, ***p<0.0001.

c. Quantification of the number of GFAP^{+} astrocytes in thalamus of Ctrl and cKO mice at different ages. n=5 mice for each group, and 3 sections from anterior to posterior of thalamus were picked up for each animal. Data are presented as mean ± SEM. Two-way ANOVA analysis followed by Tukey's post hoc analysis, *p<0.05, **p<0.001, ***p<0.0001.

d-e. The analysis of filament length (**d**), filament area (**e**), terminal points (**f**) and soma volume (**g**) of GFAP^{+} astrocytes in thalamus of Ctrl and cKO mice at different ages. n=5 mice for each group, and 3 sections were picked up from each animal. Data are presented as mean ± SEM. One-way ANOVA analysis followed by Tukey's post hoc analysis, *p<0.05, **p<0.001, ***p<0.0001.

h. Sholl analysis of GFAP^{+} astrocytes in thalamus of Ctrl and cKO mice at different ages. n=5 mice for each group, and 3 sections from the anterior, middle and posterior of thalamus were picked up for each animal. Data are presented as mean ± SEM. Two-

way ANOVA analysis followed by Tukey's post hoc analysis, *$p<0.05$, **$p<0.001$, ***$p<0.0001$.

i. Representative images for S100β and GFAP immunofluorescence staining in the thalamus of Ctrl and cKO mice at different ages. Scale bar, 20 μm.

j. Quantification of the number of S100β^+ astrocytes in thalamus of Ctrl and cKO mice at different ages. n=5 mice for each group, and 3 sections from anterior to posterior of thalamus were picked up for each animal. Data are presented as mean ± SEM. Two-way ANOVA analysis followed by Tukey's post hoc analysis, *$p<0.05$, **$p<0.001$, ***$p<0.0001$.

Supplementary Fig. 4

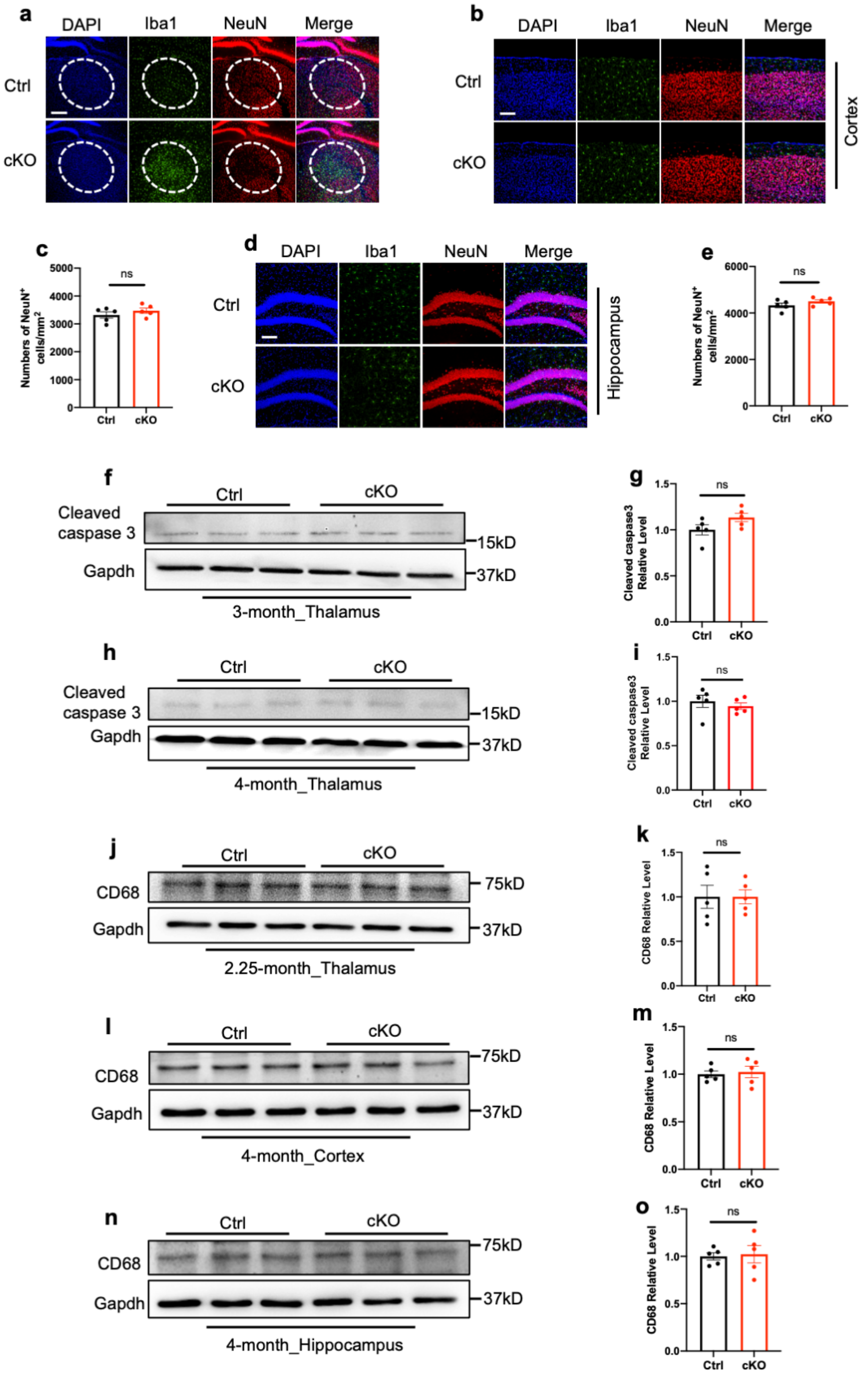

Supplementary Fig. S4. *Mettl3* deficiency leads to neuronal loss in the thalamus, but not induces apoptosis in the thalamus and neuronal loss in cortex and hippocampus.

a. Representative images for Iba1 and NeuN immunofluorescence staining in thalamus of 4-month-old Ctrl and cKO mice. Dashed lines circled the area of thalamus. Scale bar, 20 μm.

b, c. Representative images of Iba1 and NeuN (**b**) immunofluorescence staining and quantification of the number of $NeuN^{+}$ cells (**c**) in cortex of 4-month-old Ctrl and cKO mice. n=5 mice for each group, and 3 sections were picked up from each animal. Data are presented as mean ± SEM. Unpaired Student's t test, *p<0.05, **p<0.001, ***p<0.0001. Scale bar, 100 μm.

d, e. Representative images of Iba1 and NeuN (**d**) immunofluorescence staining and quantification of the number of $NeuN^{+}$ cells (**e**) in hippocampus of 4-month-old Ctrl and cKO mice. n=5 mice for each group, and 3 sections were picked up from each animal. Data are presented as mean ± SEM. Unpaired Student's t test, *p<0.05, **p<0.001, ***p<0.0001. Scale bar, 100 μm.

f, g. WB assay (**f**) and quantification results of Cleaved caspase 3 (**g**) in thalamus of 3-month-old Ctrl and cKO mice. n=5 mice for each group. Data are presented as mean ± SEM. Unpaired Student's t test, *p<0.05, **p<0.001, ***p<0.0001.

h, i. WB assay (**h**) and quantification results of Cleaved caspase 3 (**i**) in thalamus of 4-month-old Ctrl and cKO mice. n=5 mice for each group. Data are presented as mean ± SEM. Unpaired Student's t test, *p<0.05, **p<0.001, ***p<0.0001.

j, k. WB assay (**j**) and quantification results of CD68 (**k**) in thalamus of 2.25-month-old Ctrl and cKO mice. n=5 mice for each group. Data are presented as mean ± SEM. Unpaired Student's t test, *p<0.05, **p<0.001, ***p<0.0001.

l, m. WB assay (**l**) and quantification results of CD68 (**m**) in cortex of 4-month-old Ctrl and cKO mice. n=5 mice for each group. Data are presented as mean ± SEM. Unpaired Student's t test, *p<0.05, **p<0.001, ***p<0.0001.

n, o. WB assay (**n**) and quantification results of CD68 (**o**) in hippocampus of 4-month-old Ctrl and cKO mice. n=5 mice for each group. Data are presented as mean ± SEM. Unpaired Student's t test, *p<0.05, **p<0.001, ***p<0.0001.

Supplementary Fig. 5

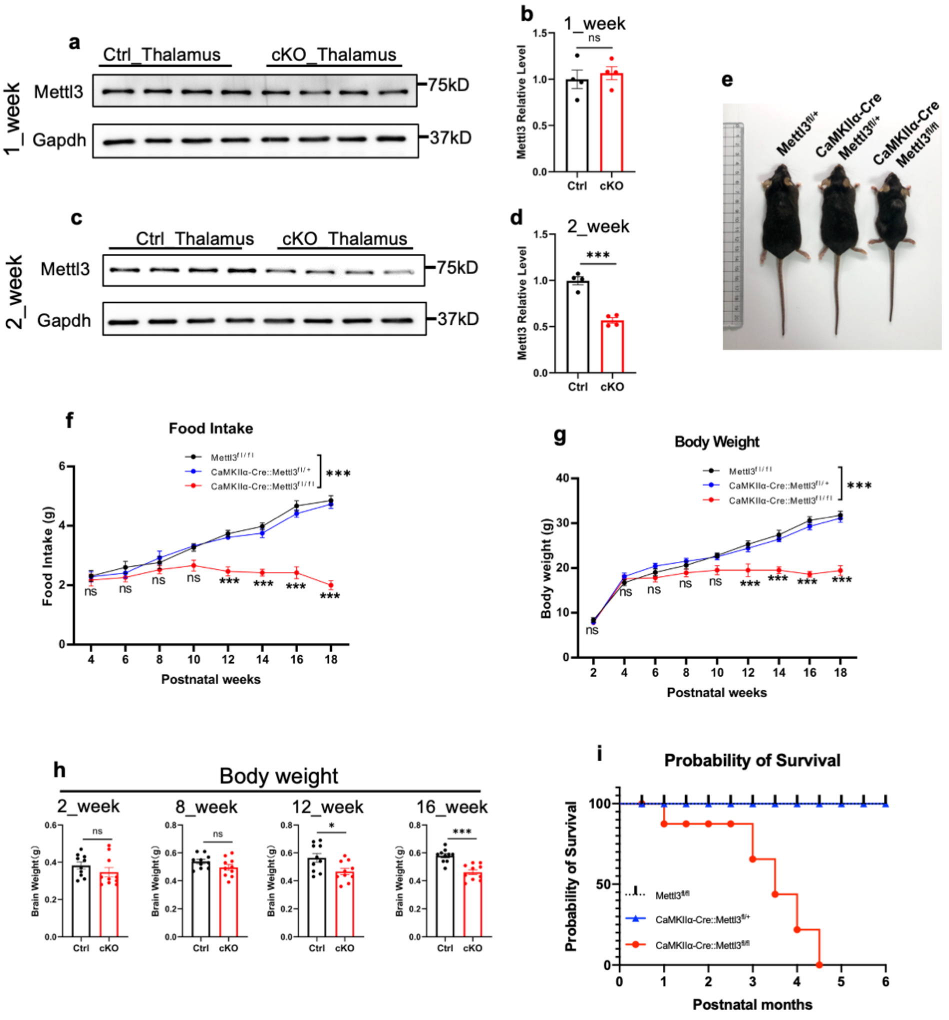

Supplementary Fig. S5. *Mettl3* deficiency leads to developmental retardation, reduces food intake, body weight, brain weight and survival rate.

a-d. Representative images of WB and quantification results of Mettl3 in thalamus of Ctrl and cKO mice at the age of 1- and 2-week.

e. Gross appearance of Ctrl, Het and *Mettl3* cKO mice at the age of 4 months.

f. Food intake analysis of Ctrl, Het and *Mettl3* cKO mice. From postnatal 4-week, animal was housed individually and the food was weighed every two weeks for each group until the age of postnatal 18 weeks. n=8 mice for each group. Two-way ANOVA analysis followed by Tukey's post hoc analysis, *p<0.05, **p<0.001, ***p<0.0001.

g, h. Body weight analysis of Ctrl, Het and *Mettl3* cKO mice. From postnatal 4-week, the body weight of animal was weighed every two weeks for each group until the age of postnatal 18 weeks. n = 8 mice per genotype. Data are presented as mean ± SEM. Two-way ANOVA analysis followed by Tukey's post hoc analysis, *p<0.05, **p<0.001, ***p<0.0001.

i. Survival curves of Ctrl, Het and *Mettl3* cKO mice, respectively. n=10 mice per genotype.

Supplementary Fig. 6

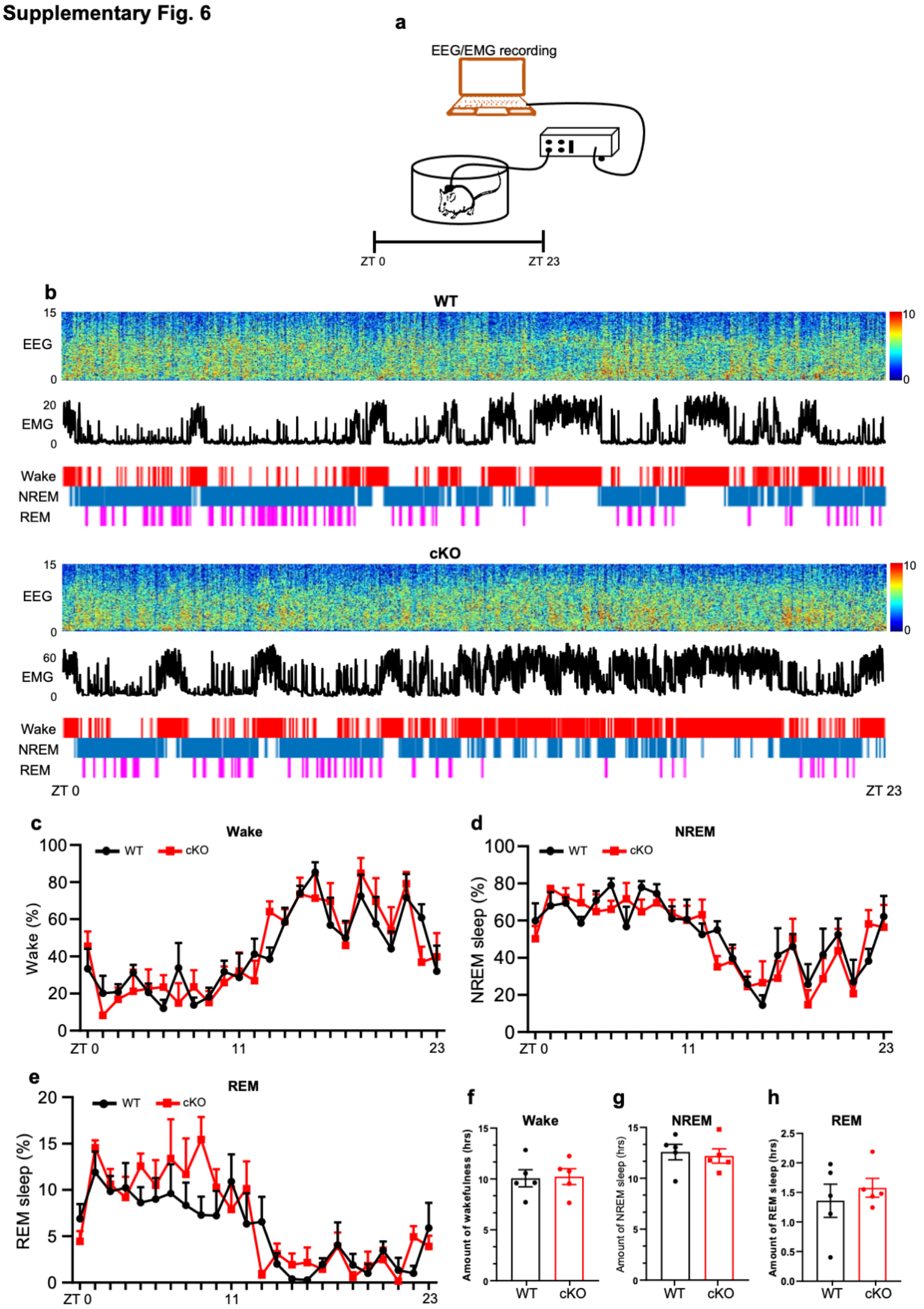

Supplementary Fig. S6. Sleep behavior assay of Ctrl and cKO mice at the age of 2-month.

a. Schematic illustration of EEG/EMG recordings.

b. Representative EEG/EMG recordings of Ctrl mice and cKO mice spanning from 8

AM (ZT 0) to 8 AM of the following day (ZT 23). Top, Representative spectrogram of EEG; Middle, EMG; Bottom, brain states annotated including wake, NREM sleep and REM sleep were shown. ZT 0 indicated the start of the light period, and ZT 12 corresponded to the beginning of darkness.

c-e. The percentage of time spent in wake (**c**), NREM sleep (**d**) and REM sleep (**e**) in each hour throughout the 24-hr recording. Data were analyzed using the two-way ANOVA with repeated measure.

f-h. The amount of time spent in wake (**f**), NREM sleep (**g**) and REM sleep (**h**) across 24-hrs recording. n=5 mice for each group. Data are presented as mean ± SEM. Unpaired Student's t test, *p<0.05, **p<0.001.

Supplementary Fig. 7

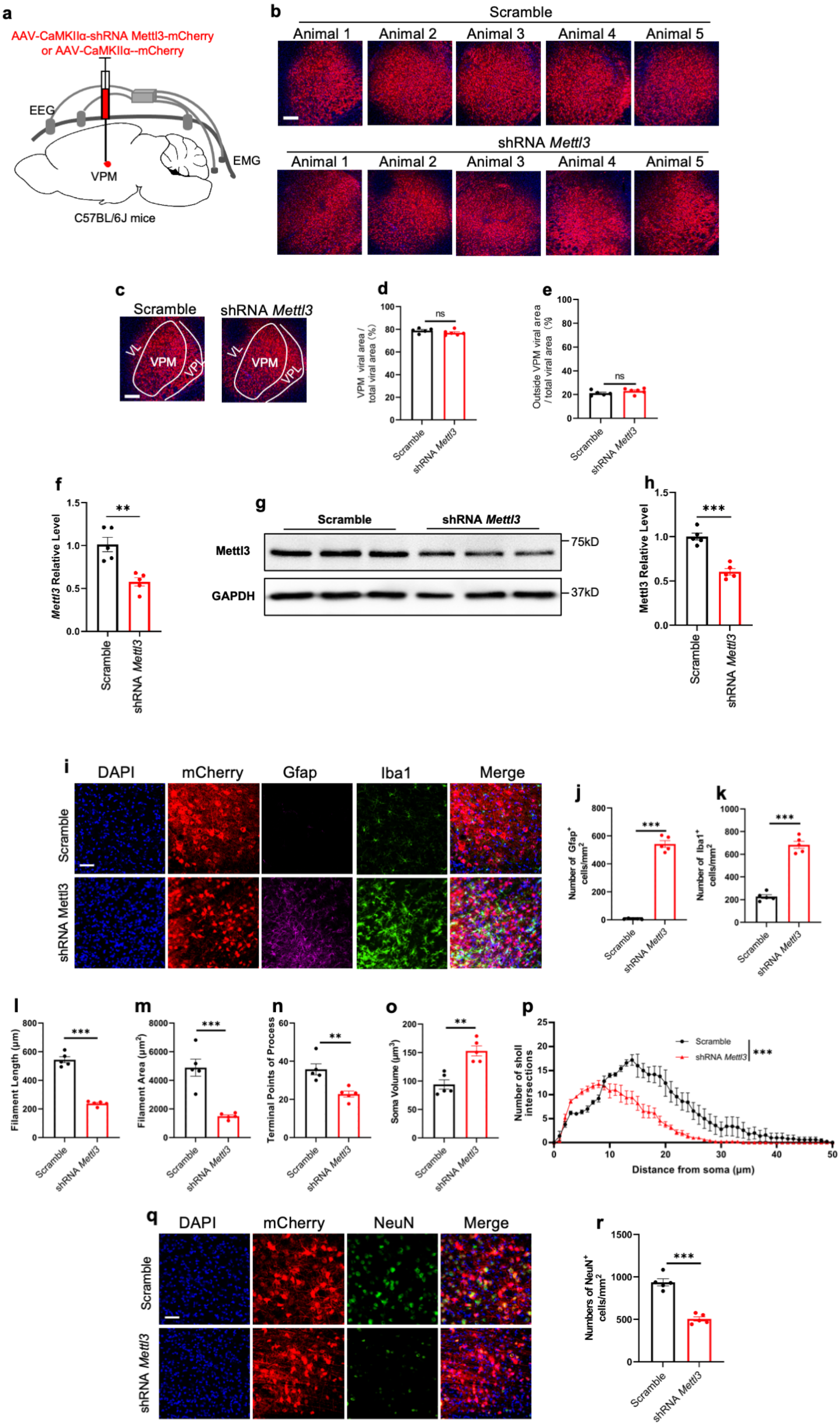

Supplementary Fig. S7. AAV-sh*Mettl3* induces high infection efficiency of VPM and reduces Mettl3 expression.

a. Schematic of AAV-scramble and AAV-shRNA against *Mettl3* injection. The bilateral thalamus of adult (8-weeks) WT C57BL/6J mice were injected with AAV-scramble and AAV-shRNA against *Mettl3*, respectively, and animals were analyzed 3 weeks post the injection.

b. Fluorescence signal indicating the infection efficiency of AAV scramble and AAV sh*Mettl3* of each animal. Scale bar, 200 μm.

c. Illustration of virus infection of VL, VPM and VPL in thalamus of mice. Scale bar, 200 μm.

d, e. Quantification of virus-infected VPM area and outside area of VPM in total virus-infected area in thalamus. n=5 mice for each group. Data are presented as mean ± SEM. Unpaired Student's t test, *p<0.05, **p<0.001, ***p<0.0001.

f. qRT-PCR assay results showed the significant decrease of *Mettl3* mRNA induced by sh*Mettl3* compared to scramble group. n=3 independent experiments for each group. Data are presented as mean ± SEM. Unpaired Student's t test, *p<0.05, **p<0.001, ***p<0.0001.

g, h. WB assay (**g**) and quantification results (**h**) showed that the level of Mettl3 was significantly decreased in sh*Mettl3* group compared to scramble group. n=5 mice for each group. Data are presented as mean ± SEM. Unpaired Student's t test, *p<0.05, **p<0.001, ***p<0.0001.

i. Representative images of mCherry-GFAP-Iba1 immunofluorescence staining in thalamus of WT mice administrated with AAV-scramble and AAV-shMettl3, respectively. Adult (8-weeks) WT mice were injected with AAV-scramble and AAV-shRNA against *Mettl3*, respectively, and were sacrificed 3 weeks post the injection. Scale bar, 25 μm.

j, k. Quantification of the number of GFAP$^+$ cells (**j**) and Iba1+ cells (**k**) in (**i**). n=5 mice for each group, and 4 sections from anterior to posterior of thalamus were picked up for each animal. Data are presented as mean ± SEM. Unpaired Student's t test, *$p<0.05$, **$p<0.001$, ***$p<0.0001$.

l-o. The analysis of filament length (**l**), filament area (**m**), terminal points of process (**n**) and soma volume of Iba1$^+$ microglia (**o**) in (**i**). n=5 mice for each group, and 3 sections were picked up from each animal. Data are presented as mean ± SEM. Unpaired Student's t test, *$p<0.05$, **$p<0.001$, ***$p<0.0001$.

p. Sholl analysis of microglia in (**i**). n=5 mice for each group, and 3 sections from the anterior, middle and posterior of thalamus were picked up for each animal. Data are presented as mean ± SEM. Two-way ANOVA analysis followed by Tukey's post hoc analysis, *$p<0.05$, **$p<0.001$, ***$p<0.0001$.

q, **r**. Representative images of mCherry-NeuN immunofluorescence staining (**q**) and quantification of the number of NeuN$^+$ cells (**r**) in thalamus of WT mice injected with AAV-scramble and AAV-sh*Mettl3*, respectively. n=5 mice for each group, and 3

sections were picked up from each animal. Data are presented as mean ± SEM. Unpaired Student's t test, *p<0.05, **p<0.001, ***p<0.0001. Scale bar, 25 μm.

Supplementary Fig. 8

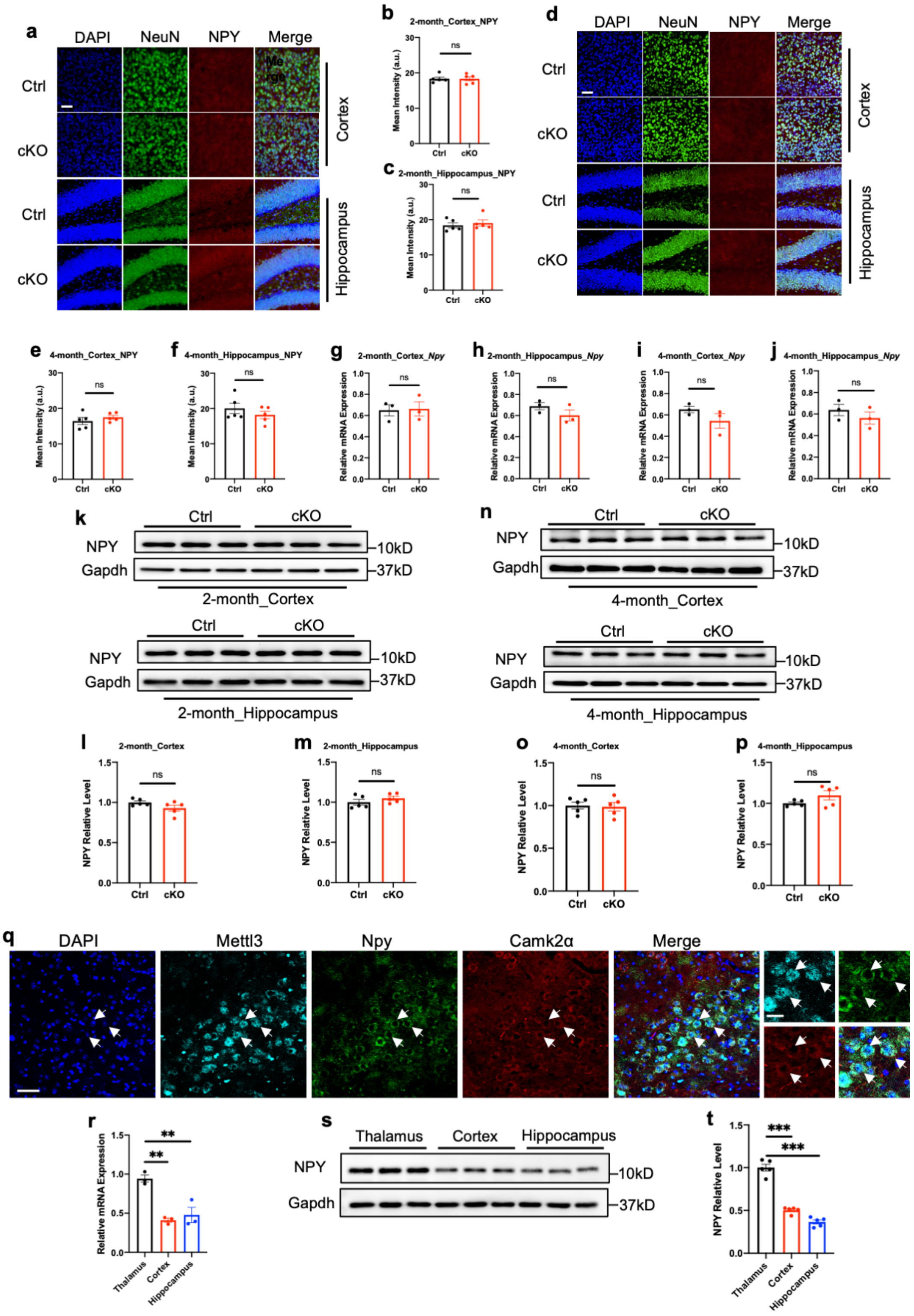

Supplementary Fig. S8. *Mettl3* deficiency does not affect the level of NPY in cortex and hippocampus.

a-c. Representative images for NeuN and NPY immunofluorescence staining and the quantification of the mean fluorescence intensity of NPY in cortex (**a**, **b**) and hippocampus (**a**, **c**) of 2-month-old Ctrl and cKO mice. n=5 mice for each group, and 3 sections were picked up from each animal. Data are presented as mean ± SEM. Unpaired Student's t test, *p<0.05, **p<0.001, ***p<0.0001. Scale bar, 50 μm.

d-f. Representative images for NeuN and NPY immunofluorescence staining and the quantification of the mean fluorescence intensity of NPY in the cortex (**d**, **e**) and hippocampus (**d**, **f**) regions of 4-month-old Ctrl and cKO mice. n=5 mice for each group, and 3 sections were picked up from each animal. Data are presented as mean ± SEM. Unpaired Student's t test, *p<0.05, **p<0.001, ***p<0.0001. Scale bar, 50 μm.

g, h. qRT-PCR assay of *Npy* mRNA expression in cortex (**g**) and hippocampus (**h**) of 2-month-old Ctrl and cKO mice. n=3 independent experiments for each group. Data are presented as mean ± SEM. Unpaired Student's t test, *p<0.05, **p<0.001, ***p<0.0001.

i, j. qRT-PCR assay of *Npy* mRNA in cortex (**I**) and hippocampus (**J**) of 4-month-old Ctrl and cKO mice. n=3 independent experiments for each group. Data are presented as mean ± SEM. Unpaired Student's t test, *p<0.05, **p<0.001, ***p<0.0001.

k-m. WB assay (**k**) and quantification results of NPY in cortex (**l**) and hippocampus (**m**) of 2-month-old Ctrl and cKO mice, respectively. n=5 mice for each group. Data

are presented as mean ± SEM. Unpaired Student's t test, *p<0.05, **p<0.001, ***p<0.0001.

n-p. WB assay (**n**) and quantification results of NPY in cortex (**o**) and hippocampus (**p**) of 4-month-old Ctrl and cKO mice, respectively. n=5 mice for each group. Data are presented as mean ± SEM. Unpaired Student's t test, *p<0.05, **p<0.001, ***p<0.0001.

q. Representative images of Mettl3-NPY-CaMKIIα immunofluorescence staining in thalamus of 2-month-old WT mice. Scale bars, 50 μm for the left panels and 25 μm for the right panels with higher magnification.

r. qRT-PCR assay of *Npy* mRNA in thalamus, cortex and hippocampus of 4-month-old Ctrl mice. n=3 independent experiments for each group. Data are presented as mean ± SEM. Unpaired Student's t test, *p<0.05, **p<0.001, ***p<0.0001.

s, t. WB assay (**s**) and quantification results of NPY in thalamus, cortex and hippocampus (**t**) of 4-month-old Ctrl. n=5 mice for each group. Data are presented as mean ± SEM. Unpaired Student's t test, *p<0.05, **p<0.001, ***p<0.0001.

Supplementary Fig. 9

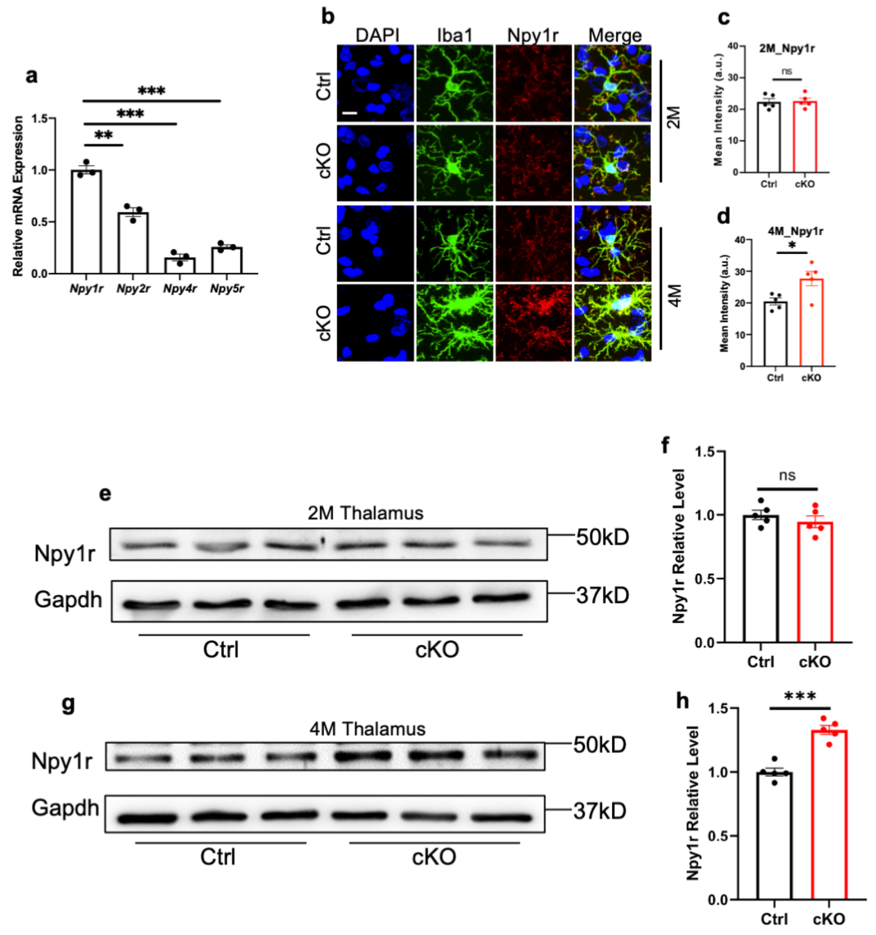

Supplementary Fig. S9. Mettl3 loss altered the expression of NPY1r in thalamus.

a. qRT-PCR assay showed that *Npy1r* had the highest expression in thalamus of adult WT mice relative to *Npy2r*, *Npy4r* and *Npy5r*. n=3 independent experiments for each group. Data are presented as mean ± SEM. Unpaired Student's t test, *p<0.05, **p<0.001, ***p<0.0001.

b-d. Representative images for Iba1 and Npy1r immunofluorescence staining (**b**) and the quantification of the mean fluorescence intensity of Npy1r in thalamus of Ctrl and

cKO mice at the age of 2-month (**c**) and 4-month (**d**), respectively. n=5 mice for each group, and 3 sections were picked up from each animal. Data are presented as mean ± SEM. Unpaired Student's t test, *p<0.05, **p<0.001, ***p<0.0001. Scale bar, 50 μm. **e-h**. WB assay and quantification results of Npy1r in thalamus of Ctrl and cKO mice at the age of 2-month (**e**, **f**) and 4-month (**g**, **h**), respectively. n=5 mice for each group. Data are presented as mean ± SEM. Unpaired Student's t test, *p<0.05, **p<0.001, ***p<0.0001.

Supplementary Fig. 10

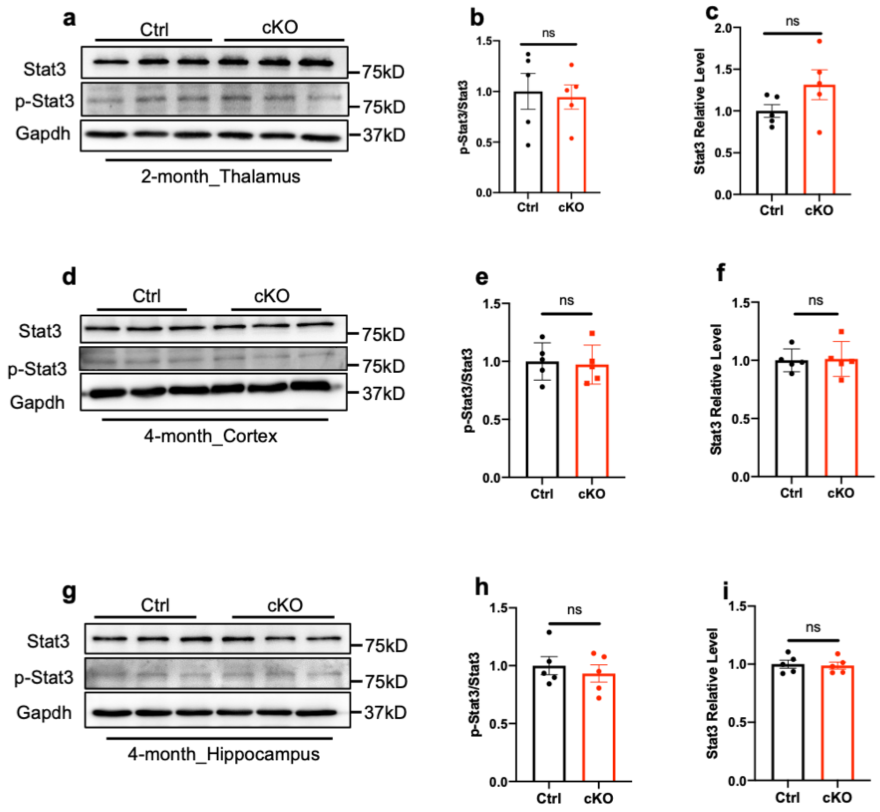

Supplementary Fig. S10. ***Mettl3*** **deficiency does not induce the activation of Stat3 pathway in cortex and hippocampus.**

a-c. WB assay (**a**) and quantification results of p-Stat3/Stat3 (**b**) and Stat3 (**c**) levels in thalamus of 2-month-old Ctrl and cKO mice, respectively. n=5 mice for each group. Data are presented as mean ± SEM. Unpaired Student's t test, *p<0.05, **p<0.001, ***p<0.0001.

d-f. WB assay (**d**) and quantification results of p-Stat3/Stat3 (**e**) and Stat3 (**f**) levels in cortex of 4-month-old Ctrl and cKO mice, respectively. n=5 mice for each group. Data are presented as mean ± SEM. Unpaired Student's t test, *p<0.05, **p<0.001, ***p<0.0001.

g-i. WB assay (**g**) and quantification results of p-Stat3/Stat3 (**h**) and Stat3 (**i**) levels in hippocampus of 4-month-old Ctrl and cKO mice, respectively. n=5 mice for each group. Data are presented as mean ± SEM. Unpaired Student's t test, *p<0.05, **p<0.001, ***p<0.0001.

Supplementary Fig. 11

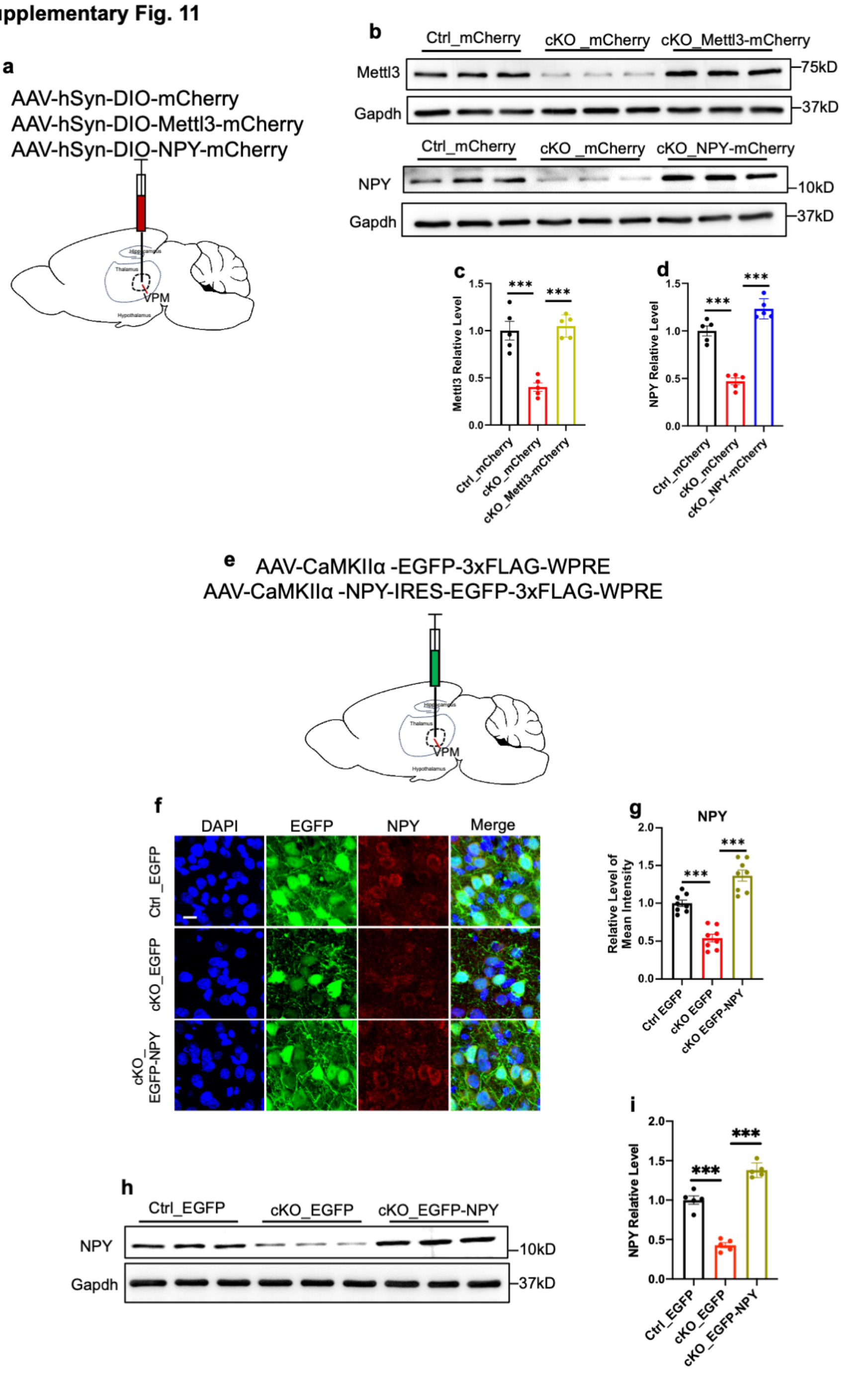

Supplementary Fig. S11. Ectopic expression of NPY restores the decreased NPY in thalamus of cKO mice.

a. Schematic of stereotactic injection of AAV-hSyn-DIO-mCherry, AAV-hSyn-DIO-Mettl3, and AAV-hSyn-DIO-NPY virus. Bilateral thalamus of 2-month-old Ctrl mice

were injected with AAV-hSyn-DIO-mCherry virus. Bilateral thalamus of 2-month-old cKO mice were injected with AAV-hSyn-DIO-mCherry, AAV-hSyn-DIO-Mettl3 and AAV-hSyn-DIO-NPY virus, respectively. 1.5-month later, animals were performed sleep behavior and biochemical assays.

b-d. WB assay (**b**) and quantification results (**c, d**) showed that AAV-Mettl3 and AAV-NPY led to significant expression of exogenous Mettl3 and NPY in thalamus of Ctrl and cKO mice 1.5-month post virus injection. n=5 mice for each group. Data are presented as mean ± SEM. One-way ANOVA analysis followed by Tukey's post hoc analysis, *$p<0.05$, **$p<0.001$, ***$p<0.0001$.

e. Schematic of stereotactic injection of AAV-CamkIIα-3xFLAG-EGFP and AAV-CamkIIα-NPY-3xFLAG-EGFP virus. Bilateral thalamus of 2-month-old Ctrl mice were injected with AAV-EGFP virus. Bilateral thalamus of 2-month-old cKO mice were injected with cKO mice were bilaterally injected AAV-EGFP and AAV-EGFP-NPY virus, respectively. 1.5-month later, animals were performed sleep behavior and biochemical assays.

f, g. Representative images for EGFP and NPY immunofluorescence staining (**f**) and the mean fluorescence intensity of NPY (**g**) in thalamus of 3-month-old Ctrl and cKO mice injected with AAV. n=5 mice for each group, and 3-5 sections were picked up from each animal. Data are presented as mean ± SEM. One-way ANOVA analysis followed by Tukey's post hoc analysis, *$p<0.05$, **$p<0.001$, ***$p<0.0001$. Scale bar, 50 μm.

h, i. WB assay (**h**) and quantification results of NPY (**i**) levels in thalamus of 3-month-old AAV-injected Ctrl and cKO mice. n=5 mice for each group. Data are presented as mean ± SEM. One-way ANOVA analysis followed by Tukey's post hoc analysis, *p<0.05, **p<0.001, ***p<0.0001.

Supplementary Fig. 12

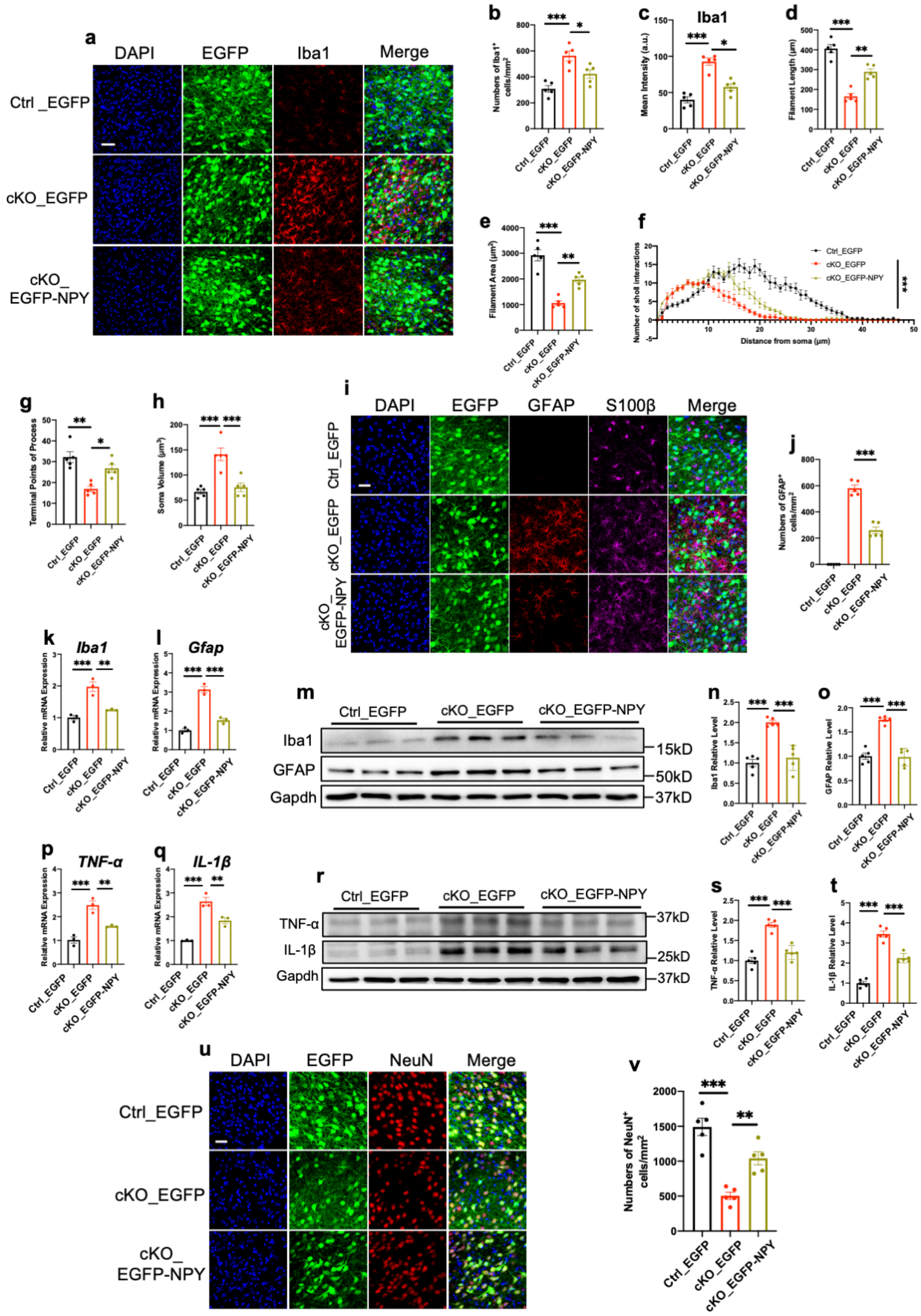

Continued Supplementary Fig. 12

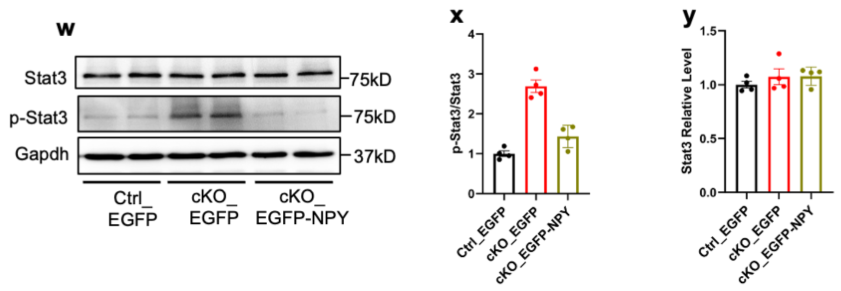

Supplementary Fig. S12. Ectopic Mettl3 and NPY inhibits neuroinflammation and neuronal loss in *Mettl3* deficient mice.

a-c. Representative images of EGFP and Iba1 (**a**) immunofluorescence staining, the quantification of the number of Iba1^{+} cells (**b**) and the mean fluorescence intensity of Iba1 (**c**) in thalamus region of Ctrl and cKO mice injected with AAV. AAV virus was injected at the age of 2-month, and mice were sacrificed 1.5 month later. n=5 mice for each group, and 3 sections were picked up from each animal. Data are presented as mean ± SEM. One-way ANOVA analysis followed by Tukey's post hoc analysis, *p<0.05, **p<0.001, ***p<0.0001. Scale bar, 50 µm.

d-h. Quantification of microglial filament length (**d**), filament area (**e**), sholl analysis (**f**), terminal points of process (**g**), and soma volume (**h**) in (**a**). n=5 mice for each group, and 3 sections were picked up from each animal. Data are presented as mean ± SEM. One-way ANOVA analysis followed by Tukey's post hoc analysis for (**d**), (**e**), (**g**) and (**h**), *p<0.05, **p<0.001, ***p<0.0001. Two-way ANOVA analysis followed by Tukey's post hoc analysis for (**F**), *p<0.05, **p<0.001, ***p<0.0001.

i, j. Representative images for EGFP, GFAP and S100β (**i**) immunofluorescence staining and the quantification of the number of $GFAP^+$ cells (**j**) in thalamus of Ctrl and cKO mice. AAV virus was injected at the age of 2-month, and mice were sacrificed 1.5 month later. n=5 mice for each group, and 3 sections were picked up from each animal. Data are presented as mean ± SEM. One-way ANOVA analysis followed by Tukey's post hoc analysis, *p<0.05, **p<0.001, ***p<0.0001. Scale bar, 50 μm.

k-m. WB assay (**k**) and quantification results of Iba1 (**l**) and GFAP (**m**) levels in thalamus of Ctrl and cKO mice. AAV virus was injected at the age of 2-month, and mice were sacrificed 1.5 month later. n=5 mice for each group. Data are presented as mean ± SEM. One-way ANOVA analysis followed by Tukey's post hoc analysis, *p<0.05, **p<0.001, ***p<0.0001.

n, o. qRT-PCR assay of Iba1 (**n**) and Gfap (**o**) mRNA expression in thalamus of AAV-injected Ctrl and cKO mice. Mice were injected with AAV virus at the age of 2-month, and sacrificed 1.5 month later. n=3 independent experiments for each group. Data are presented as mean ± SEM. One-way ANOVA analysis followed by Tukey's post hoc analysis, *p<0.05, **p<0.001, ***p<0.0001.

p-r. WB assay (**p**) and quantification results of TNF-α (**q**) and IL-1β (**r**) levels in thalamus of AAV-injected Ctrl and cKO mice. Mice were injected with AAV virus at the age of 2-month, and sacrificed 1.5 month later. n=5 mice for each group. Data are presented as mean ± SEM. One-way ANOVA analysis followed by Tukey's post hoc analysis, *p<0.05, **p<0.001, ***p<0.0001.

s, t. qRT-PCR assay of TNF-α (**s**) and IL-1β (**t**) mRNA expression in thalamus of AAV-injected Ctrl and cKO mice. Mice were injected with AAV virus at the age of 2-month, and sacrificed 1.5 month later. n=3 independent experiments for each group. Data are presented as mean ± SEM. One-way ANOVA analysis followed by Tukey's post hoc analysis, *p<0.05, **p<0.001, ***p<0.0001.

u, v. Representative images for EGFP and NeuN (**u**) immunofluorescence staining and the quantification of the number of NeuN$^+$ cells (**v**) in thalamus regions of AAV-injected Ctrl and cKO mice. Mice were injected with AAV virus at the age of 2-month, and sacrificed 1.5 month later. n=5 mice for each group, and 3 sections were picked up from each animal. Data are presented as mean ± SEM. One-way ANOVA analysis followed by Tukey's post hoc analysis, *p<0.05, **p<0.001, ***p<0.0001. Scale bar, 50 μm.

w-y. WB assay (**w**) and quantification results of p-Stat3 (**x**) and Stat3 (**y**) levels in thalamus of AAV-injected Ctrl and cKO mice. Mice were injected with AAV virus at the age of 2-month, and sacrificed 1.5 month later. n=4 mice for each group. Data are presented as mean ± SEM. One-way ANOVA analysis followed by Tukey's post hoc analysis, *p<0.05, **p<0.001, ***p<0.0001.

Supplementary Fig. 13

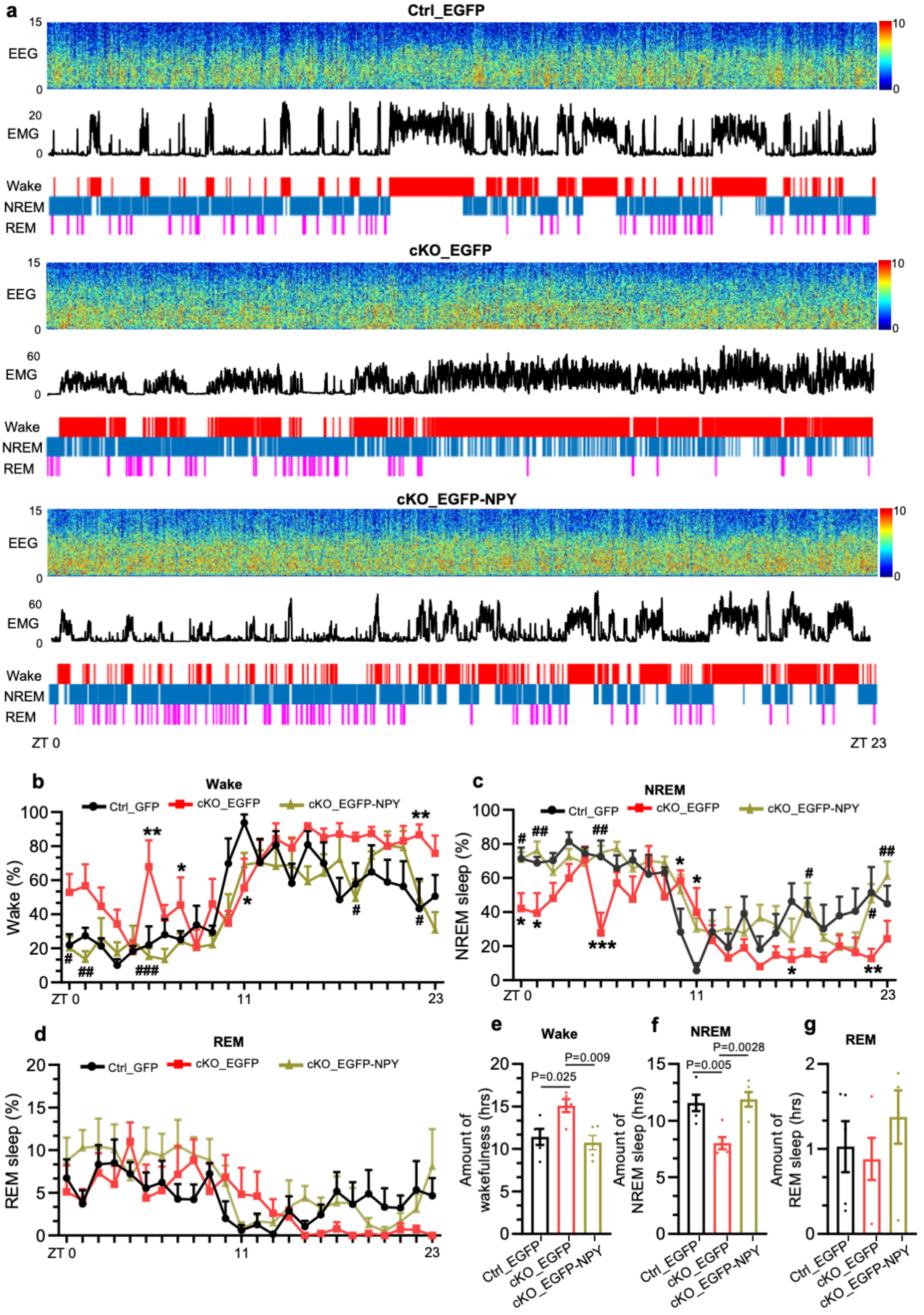

Supplementary Fig. S13. Ectopic NPY restores the disrupted sleep behavior of *Mettl3* deficient mice.

a. Representative EEG/EMG recordings of Ctrl mice with AAV-EGFP injection (Ctrl_EGFP group), and cKO mice with AAV_EGFP (cKO_EGFP) or AAV_EGFP-NPY (cKO_EGFP-NPY group) injection spanning 8 AM (ZT 0) to 8 AM of the following day (ZT 23). Top, Representative spectrogram of EEG; Middle, EMG; Bottom, brain states annotated including wake, NREM sleep and REM sleep were shown. ZT 0 indicated the start of the light period, and ZT 12 corresponded to the beginning of darkness.

b-d. The percentage of time spent in wake (**b**), NREM sleep (**c**) and REM sleep (**d**) in each hour throughout the 24-hr recording. Data were analyzed using the two-way ANOVA with repeated measure.

e-g. The amount of time spent in wake (**e**), NREM sleep (**f**) and REM sleep (**g**) across 24-hrs recording. n=5 mice for each group. Data are presented as mean ± SEM. Data are presented as mean ± SEM. One-way ANOVA analysis followed by Tukey's post hoc analysis.
